# Co-delivery of camptothecin and MiR-145 by lipid nanoparticles for MRI-visible targeted therapy of hepatocellular carcinoma

**DOI:** 10.1186/s13046-024-03167-9

**Published:** 2024-08-30

**Authors:** Jing Rong, Tongtong Liu, Xiujuan Yin, Min Shao, Kun Zhu, Bin Li, Shiqi Wang, Yujie Zhu, Saisai Zhang, Likang Yin, Qi Liu, Xiao Wang, Lei Zhang

**Affiliations:** 1 Department of Radiology, The First Affiliated Hospital of Anhui Medical University, Anhui Medical University, Hefei, 230022 China; 2 School of Pharmacy, Key Laboratory of Anti-inflammatory of Immune Medicines of Ministry of Education, Inflammation and Immune Mediated Diseases Laboratory of Anhui Province, Anhui Institute of Innovative Drugs, Anhui Medical University, Hefei, 230032 China

**Keywords:** Combination therapy, Lipid nanoparticles, Target delivery, Magnetic resonance imaging, Hepatocellular carcinoma

## Abstract

**Background:**

Camptothecin (CPT) is one of the frequently used small chemotherapy drugs for treating hepatocellular carcinoma (HCC), but its clinical application is limited due to severe toxicities and acquired resistance. Combined chemo-gene therapy has been reported to be an effective strategy for counteracting drug resistance while sensitizing cancer cells to cytotoxic agents. Thus, we hypothesized that combining CPT with miR-145 could synergistically suppress tumor proliferation and enhance anti-tumor activity.

**Methods:**

Lactobionic acid (LA) modified lipid nanoparticles (LNPs) were developed to co-deliver CPT and miR-145 into asialoglycoprotein receptors-expressing HCC in vitro and in vivo. We evaluated the synergetic antitumor effect of miR-145 and CPT using CCK8, Western blotting, apoptosis and wound scratch assay in vitro, and the mechanisms underlying the synergetic antitumor effects were further investigated. Tumor inhibitory efficacy, safety evaluation and MRI-visible ability were assessed using diethylnitrosamine (DEN) + CCl_4_-induced HCC mouse model.

**Results:**

The LA modification improved the targeting delivery of cargos to HCC cells and tissues. The LA-CMGL-mediated co-delivery of miR-145 and CPT is more effective on tumor inhibitory than LA-CPT-L or LA-miR-145-L treatment alone, both in vitro and in vivo, with almost no side effects during the treatment period. Mechanistically, miR-145 likely induces apoptosis by targeting SUMO-specific peptidase 1 (SENP1)-mediated hexokinase (HK2) SUMOylation and glycolysis pathways and, in turn, sensitizing the cancer cells to CPT. In vitro and in vivo tests confirmed that the loaded Gd-DOTA served as an effective T1-weighted contrast agent for noninvasive tumor detection as well as real-time monitoring of drug delivery and biodistribution.

**Conclusions:**

The LA-CMGL-mediated co-delivery of miR-145 and CPT displays a synergistic therapy against HCC. The novel MRI-visible, actively targeted chemo-gene co-delivery system for HCC therapy provides a scientific basis and a useful idea for the development of HCC treatment strategies in the future.

**Supplementary Information:**

The online version contains supplementary material available at 10.1186/s13046-024-03167-9.

## Background

Hepatocellular carcinoma (HCC) is the third most frequent cause of cancer‑associated deaths worldwide, accounting for over 90% of liver cancer cases [1]. Systematic chemotherapy is currently the primary treatment for HCC [2, 3]. Camptothecin (CPT), an inhibitor of topoisomerase I, is one of the frequently used small chemotherapy drugs for treating patients with solid malignant tumors, including HCC [4, 5]. However, these drugs have poor solubility in water and generate a high amount of toxicity and side effects, compromising patients’ quality of life [6, 7]. Moreover, HCC often develops resistance to these drugs due to the one-dimensional action mechanism of single drug therapy [8, 9]. Therefore, there is an urgent need for the development an effective therapeutic strategy for clinical use.

MicroRNA (miRNA or miR) refers to a group of endogenous, noncoding small RNA that regulate a significant number of proteins at the translation level, making their use a popular gene therapy technique for cancer treatment [10]. Notably, miR-145, a member of the miRNA family, has been found to downregulate numerous malignant tumors and identified as a critical suppressor of cancer initiation, metastasis, and therapeutic resistance [11–14]. Moreover, overexpression of miR-145 can reduce the extracellular acidification rate (ECAR) and suppress glycolysis in various cancer cells by regulating the aerobic glucose metabolism [12, 14]. ECAR are widely used proxies for glycolytic rate in cell metabolism studies and an increase in ECAR may indicate a greater reliance on glycolysis by the cells [15]. Despite the critical role of miR-145 in cancer cell regulation, it has not been sufficiently explored as a therapeutic target for HCC, either through monotherapy or combination therapy [12, 16]. Therefore, it is necessary to explore the anti-HCC pathway of miR-145 and pay more attention to its potential for clinical application.

Combined chemo-gene therapy has been reported to be an effective strategy for counteracting drug resistance while sensitizing cancer cells to cytotoxic agents [7, 17–19], thereby addressing the urgent need for nonsurgical therapy for liver cancer [6, 20]. However, finding an effective and safe delivery strategy is the main challenge affecting the successful clinical translation of combined therapy [10, 21]. Lipid nanoparticles (LNPs) containing an ionizable lipid (DLin-MC3-DMA, MC3) are currently among the most advanced oligonucleotide delivery systems [22–24]. MC3 is essential for RNA therapeutics protection and for driving lysosomal escape due to its ability to acquire charge after endocytosis [23, 25, 26]. In 2018, the first MC3-based siRNA therapeutic drug, Onpattro, was approved by the US Food and Drug Administration for the treatment of transthyretin-mediated amyloidosis [27]. More recently, a novel strategy named selective organ targeting (SORT) has been reported, which allows MC3-based LNPs containing four components to be systematically designed for the accurate delivery of diverse RNA to the livers of mice following intravenous (IV) administration [25, 28]. Given the significant progress in the development of LNP-based nucleic acid delivery systems, we are positive that LNPs provide the most promising delivery platforms for the intravenous co-delivery of miRNA and small chemotherapeutics in HCC therapy [18, 29, 30].

Gd-DOTA is a rather safe MRI contrast agent that has been widely applied in clinical settings [31, 32]. The introduction of Gd^3+^ into nanosystems enables the MRI-visible delivery and biodistribution of drug carriers as well as cancer diagnosis [5, 32, 33]. In traditional medical care, diagnosis and therapy are considered two separate issues [2, 34]. However, mounting evidence has shown that combining them mutually and synergically allows for achieving optimal personalized curative effects for many cancers [31, 35]. Targeted delivery is particularly important in the treatment of HCC because it helps decrease systemic toxicity and off-target effects and promotes the accumulation of drugs and contrast agents in tumor cells and tissues [35, 36]. Asialoglycoprotein receptors (ASGPRs) are cell membrane receptors that are overexpressed on the surface of liver cancer cells [6, 37]. Lactobionic acid (LA), which comprises gluconic acid and Gal moiety, has been extensively investigated as a liver cancer-targeting ligand due to its specific affinity to ASGPRs [35, 38]. Therefore, the incorporation of LA into LNPs would enhance the targeted delivery of cargos in liver cancer tissue *via* ASGPR-mediated endocytosis [39].

**Scheme 1:**
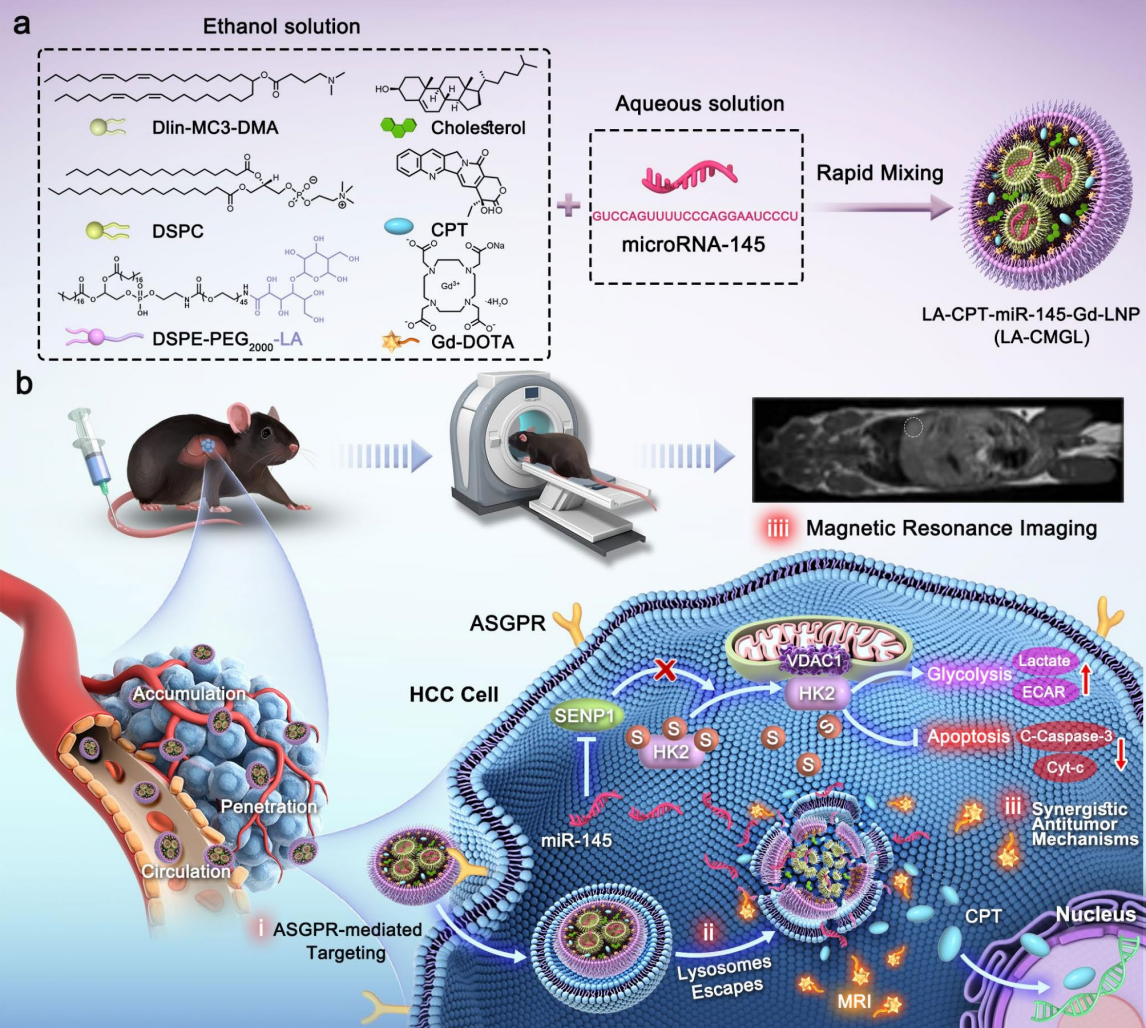
Schematic illustration for the design and application of LA-CMGL in HCC therapy. (**a**) Formulation of LA-CMGL by rapid pipette mixing of acidic aqueous solution and ethanol solution. (**b**) Schematic illustration of LA-CMGL co-delivery of CPT/miR-145 and Gd-DOTA to achieve MRI-visible targeted therapy of HCC (i) ASGPR-mediated targeting. (ii) Lysosome escape. (iii) Synergetic antitumor mechanism of CPT and miR-145, with the released CPT inducing the apoptosis of HepG2 cells by binding to chromosomal DNA and miR-145 promoting HepG2 cells apoptosis via the SENP1-mediated HK2 SUMOylation and glycolysis pathways. iiii) MR targeted imaging and real-time monitoring of drug delivery

In this study, we fabricated LA-modified MC3-based LNPs for the co-delivery of CPT/miR-145 and Gd-DOTA to simultaneously achieve combined chemo-gene therapy for HCC and real-time MR imaging (Scheme 1). The LNPs protected miR-145 from degradation by endo/exonucleases and improved the water insolubility of CPT. After internalization *via* ASGPR-mediated endocytosis, the LA-modified CPT/miR-145 and Gd-DOTA coloaded lipid nanoparticles (LA-CMGL) disassembled rapidly in the acidic lysosomal environment and led to lysosomal escape due to the ion-pair mechanism [27, 40]. The incorporated cargos, namely CPT/miR-145 and Gd^3+^, were then released into the cytoplasm. CPT/miR-145 in the LA-CMGL showed excellent synergetic antitumor effects both in vitro and in a diethylnitrosamine (DEN) + CCl_4_-induced HCC mouse model. The loaded Gd^3+^ served as an effective T1-weighted contrast agent for noninvasive tumor detection and the real-time monitoring of drug delivery. In addition, a new mechanism for inhibition of HCC growth by LA-CMGL was demonstrated: specifically, miR-145 sensitizes cancer cells to CPT and promotes apoptosis by targeting the SENP1-mediated HK2 SUMOylation and glycolysis pathways. These findings open up a new avenue for the design of nano-based medicines for personalized cancer therapy.

## Methods

### Materials

DLin-MC3-DMA (heptatriaconta-6,9,28,31-tetraen-19-yl-4[dimethylamino]-butanoa-te), DSPC (1,2-distearoyl-sn-glycero-3-phosphocholine), cholesterol, LA (lactobio-nic acid), and DSPE-PEG2000-NH2 (1,2-distearoyl-sn-glycero-3-Phosphoethanola-mine-N-[methoxypolyethylene glycol-2000]) were obtained from Shanghai Yua Ye Bio-Technology Co., Ltd. CPT was purchased from Sigma-Aldrich. Cy5.5-miRNA-145 (abbreviated as miR-145 unless otherwise stated) was purchased from Shanghai GenePharma Co., Ltd. Gd-DOTA was purchased from Xi’an Kaixin Biotechnology Co., Ltd. FBS was obtained from Thermo Fisher Scientific, USA. All chemical reagents and solvents were of commercial special grade and were used without further purification.

### Preparation of LA-CMGL

DSPE-PEG-LA was prepared by conjugating LA onto DSPE-PEG2000-NH2. Specifically, LA (53.7 mg, 150 µmol), EDC (43 mg, 225 µmol), and NHS (26 mg, 225 µmol) were dissolved in PBS (pH 7.4, 10 mM), and the mixture was stirred for 2 h at 0℃. DSPE-PEG2000-NH2 (140 mg, 50 µmol) was then added, and the mixture was stirred for another 12 h. After the reaction, the unreacted LA was removed *via* dialysis (MWCO = 3000 Da) against distilled water, and DSPE-PEG-LA was obtained by lyophilization. The successful synthesis of DSPE-PEG-LA was confirmed using ^1^H NMR analysis.

LA-CPT-miR-145-Gd-LNP (abbreviated as LA-CMGL) was prepared using an ethanol dilution method. To this end, 2 mM ionizable lipid DLin-MC3-DMA was dissolved in ethanol with 1 mM DSPC, 1 mM Chol, and 0.5 mM DSPE-PEG-LA to prepare a lipid ethanol solution with the same molar composition (50/10/38.5/1.5, mol/mol) as the Onpattro formulation [27, 28]. CPT and Gd-DOTA were also dissolved in ethanol, and miR-145 was dissolved in citrate buffer (10 mM, pH 4.0). The ethanol solution (40 µL) was then rapidly mixed with miR-145 solution (120 µL) at a volume ratio of 1:3 and a w/w ratio of 40:1 (lipid/miR-145). To achieve a final weight ratio of 40:1 for total lipid / total RNA, the concentration of miR-145 was set as 0.86 µg/µL. The dosages of CPT and Gd-DOTA were 15 µmol/kg and 40 µmol/kg, respectively. After 10 min of incubation at room temperature, the formulated LNPs were diluted with 1 × PBS for the in vitro studies. For in vivo studies, the LNPs were further purified by dialysis in sterile 1 × PBS dialysis tubes (MWCO = 3500 Da) for 2 h. Blank and single drug-loaded LNPs were prepared and modified using the same conditions as above and labeled blank L, CPT-L, miR-145-L, LA-CPT-L, and LA-miR-145-L. CMGL was prepared without any LA modification using the same method.

### Characterization of lipid nanoparticles

Dynamic light scattering (DLS) was performed using the zetasizer Nano ZS (Malvern Panalytical, Malvern, UK) to determine the size distribution and zeta potential of LA-CMGL, CMGL, and blank L. The morphologies of the LNPs were observed using transmission electron microscopy (TEM, Thermo Fisher Scientific, USA). The encapsulation efficiencies (EE%) of CPT and miR-145 were determined using a fluorescence spectrophotometer (Spectrofluorometer FS5, Edinburgh Instruments Ltd, UK) [7, 40]. The Gd^3+^ content loaded in LA-CMGL was determined using an inductively coupled plasma-optical emission spectrometer (ICP-OES, PerkinElmer Optima 7000 DV, USA).

### In vitro stability and drug release

To explore the stability of the LA-CMGL, their sizes and polydispersity indexes (PDIs) were monitored using DLS for one week, and they were stored in PBS (4 °C and 37 °C). To evaluate the protective ability of LA-CMGL against miR-145, free miR-145 and LA-CMGL were incubated in mouse serum for 2, 6, 8, 12, and 24 h, and the integrity of miR-145 was analyzed using a gel retardation assay.

The release profiles of LA-CMGL in PBS with different pH values (pH 4.5, pH 6.5, and pH 7.4) were evaluated using the dialysis method. Specifically, 1 mL samples of free CPT and LA-CMGL were dispersed separately and transferred immediately to a dialysis bag (MWCO = 5000 Da) against 20 mL of PBS at 37 °C. At predetermined time points (0, 2, 4, 6, 12, 24, 48, 72, and 96 h), 2 mL of the release medium was collected, and an equal volume of fresh PBS was added. The quantities of released CPT were measured using a fluorescence spectrophotometer.

### Cellular uptake and lysosomal escape

For the cellular internalization study, we selected two cell lines: ASGPR-overexpressed HepG2 cancer cells and ASGPR-underexpressing HepaRG cells (Figure S1). HepaRG cells share some features and properties with adult liver cells, making them particularly useful for evaluating drugs [41]. Each cell type was seeded in 24-well plates (1 × 10^5^ cells/well) and cultured overnight at 37 ℃. The culture medium was then refreshed with a serum-free Opti-MEM medium containing LA-CMGL or CMGL loaded with Cy5.5-miR-145 (50 nM) at 37 ℃. After 6 h of incubation, the cells were collected, washed with cold PBS thrice, and analyzed *via* confocal laser scanning microscopy (CLSM, Zeiss, LSM-700) and flow cytometry (Beckman, CytoFLEX, USA). For the competition assay, 1 mM of free LA was added to the incubation media prior to the addition of LA-CMGL, followed by the same steps described above. All experiments were performed in triplicate.

The intracellular delivery and distribution of Cy5.5-miR-145 were investigated through CLSM after cellular internalization. HepG2 cells were incubated with LA-CMGL for 1 h and 6 h. At the preassigned 30 min time point, 1 mL LysoTracker Green (1 mM) was added to stain the lysosomes, and lysosomal escape was observed using CLSM. The degree of Cy5.5-miR-145 and Lyso-Tracker Green colocalization was assessed by Image J.

### In vitro cytotoxicity, apoptosis and cell migration assays

The cytotoxic effects of various formulations against HepG2 cells or HepaRG cells were assessed using a Cell Counting Kit-8 (CCK8, Biosharp, Japan). In brief, HepG2 or HepaRG cells were seeded in 96-well plates at a density of 1 × 10^4^ per well at 37 °C. First, HepG2 cells were treated with different doses of CPT for 24 h. Second, HepG2 cells were treated with fixed concentrations of free drugs (CPT, miR-145, and CPT + miR-145) at 37 °C for 60 h; CPT and miRNA concentrations were 10 µg/mL and 100 nM, respectively. Third, HepG2 or HepaRG cells were treated with the same fixed concentrations of LA-NC-L, LA-CPT-L, LA-miR-145-L, LA-CMGL, and CMGL at 37 ℃ for 24 h. The cells treated with PBS were used as controls. Each section of the assay was performed in triplicate. Cell viability was determined using the CCK8 kit in accordance with the manufacturer’s suggestions.

To confirm the anticancer effect of the CPT/miR-145 coloaded LNPs, a cell apoptosis assay was performed using the annexin V-FITC/PI staining method. Briefly, HepG2 cells were seeded into six-well plates at a density of 1 × 10^5^ cells per well and incubated overnight at 37 ℃. These cells were then treated with LA-NC-L, LA-CPT-L, LA-miR145-L, LA-CMGL, and CMGL in serum-free Opti-MEM at 37 ℃ for 48 h. Cells treated with PBS were used as controls. An Annexin V-FITC Apoptosis Detection Kit (Biosharp, China) was used to determine the apoptosis rate according to the manufacturer’s protocol.

To evaluate cell migration inhibition ability of LA-CMGL, the wound scratch assay was carried out. HepG2, Huh7 and Hep3B cells were seeded in 6-well plates at a density of 5 × 10^5^/ well. When the cell reached 80–90% confluent monolayer, the cells were scratched off the plate with 200 µL pipette tip and washed with PBS. Then, the cells were treated with LA-NC-L, LA-CPT-L, LA-miR-145-L, CMGL and LA-CMGL. Cells without any treatment served as controls. Eventually, photographs were taken with a Zeiss microscope (Oberkochen, German) at 0 h and 24 h. The areas of the scratch were quantified by the Image J software and the wound closure rate = (0 h area − 24 h area/ 0 h area × 100%.

### In vitro MRI

The longitudinal relaxation rate (1/T1) values for LA-CMGL and Gd-DOTA were measured using an Ingenia 1.5T MRI and calculated in line with our previous report [5]. Next, HepG2 cells and HepaRG cells were incubated in LA-CMGL with various Gd^3+^ concentrations at 37 ℃ for 12 h. After washing them in a plate with 5 mL of PBS three times, the cells were transferred to microtubes for cellular MRI.

### Antitumor effects and safety evaluation of LA-CMGL in vivo

To evaluate the antitumor effects of LA-CMGL, the DEN + CCl_4_-induced HCC mouse model was established as described [42, 43]. The mice were randomly divided into six groups (*n* = 12 per group) and received intravenous administration of PBS (control group), LA-NC-L, LA-CPT-L, LA-miR-145, CMGL, or LA-CMGL with the final CPT concentration of 10 µg/mL and miR-145 concentration of 100 mM. The different formulations (100 µL) were administered intravenously twice per week from week 28 to 36. The mice’s body weights were recorded every other day since the treatment began. In the 36th week after treatment, blood was collected, the alanine transaminase (ALT) and aspartate aminotransferase (AST) levels were analyzed to assess liver toxicity with the different formulations. The ALT and AST levels of healthy mice treated with PBS were used as controls (*n* = 3). The model mice were sacrificed, and the highest tumor volume and tumor number were recorded (*n* = 5 per group). For histological examinations, the tumor tissues and main organs (heart, liver, spleen, lung, and kidney) were removed. The biggest tumor tissue was selected and fixed in 4% paraformaldehyde at 4 °C overnight and later embedded in paraffin. Tumor sections of 5 μm were then prepared for H&E or TUNEL staining and microscopic observation. Part of the tumor tissue was homogenized in RIPA buffer (Thermo Scientific), and proteins were extracted for a WB analysis of apoptotic protein expression, as described above. The major organs were also stained with H&E for pathological analysis. The survival times of the mice were recorded daily from the beginning of treatment to the day of death and analyzed *via* a Kaplan − Meier survival study (*n* = 7 per group).

### MRI-visible targeted delivery of LA-CMGL in vivo

DEN + CCl_4_-induced HCC model mice were studied to evaluate the in vivo biodistribution and tumor-targeting MR imaging capabilities of LA-CMGL (*n* = 3). Free Gd-DOTA, CMGL, or LA-CMGL were injected at a dose of 40 µmoL Gd/kg through the tail veins of the HCC model mice. The mice were anesthetized with an intraperitoneal injection of 1% sodium pentobarbital at a dose of 50 mg/kg. Precontrast and postcontrast T1-weighted magnetic resonance images were acquired using a 3.0T scanner (GE Signa Horizon) with a small animal coil and a fast spin-echo pulse sequence. The parameters were as follows: TR/TE = 400/12 ms, FOV 8 × 6.4 cm^2^, matrix 256 × 192, slices/space 1.0/0.5 mm, and NEX 6–8. To quantitatively analyze the biodistribution of LA-CMGL, the contrast-to-noise ratio (CNR) of a specific organ was computed using the following equation: CNR = Sp-S0/Sn, where Sp (post-injection) and S0 (pre-injection) denote the signal intensity in the region of interest (ROI), and Sn is the standard deviation of noise estimated from the background air. In addition, the HCC-targeting MRI capability of LA-CMGL was evaluated by calculating the T/N ratio using the same experimental procedure as described above. T/N ratios represent the signal intensities of various Gd^3+^ preparations within the regions of interest of tumor and normal liver tissues.

## Results and discussion

### Synthesis and characterization of DSPE-PEG-LA and LA-CMGL

**Fig. 1:**
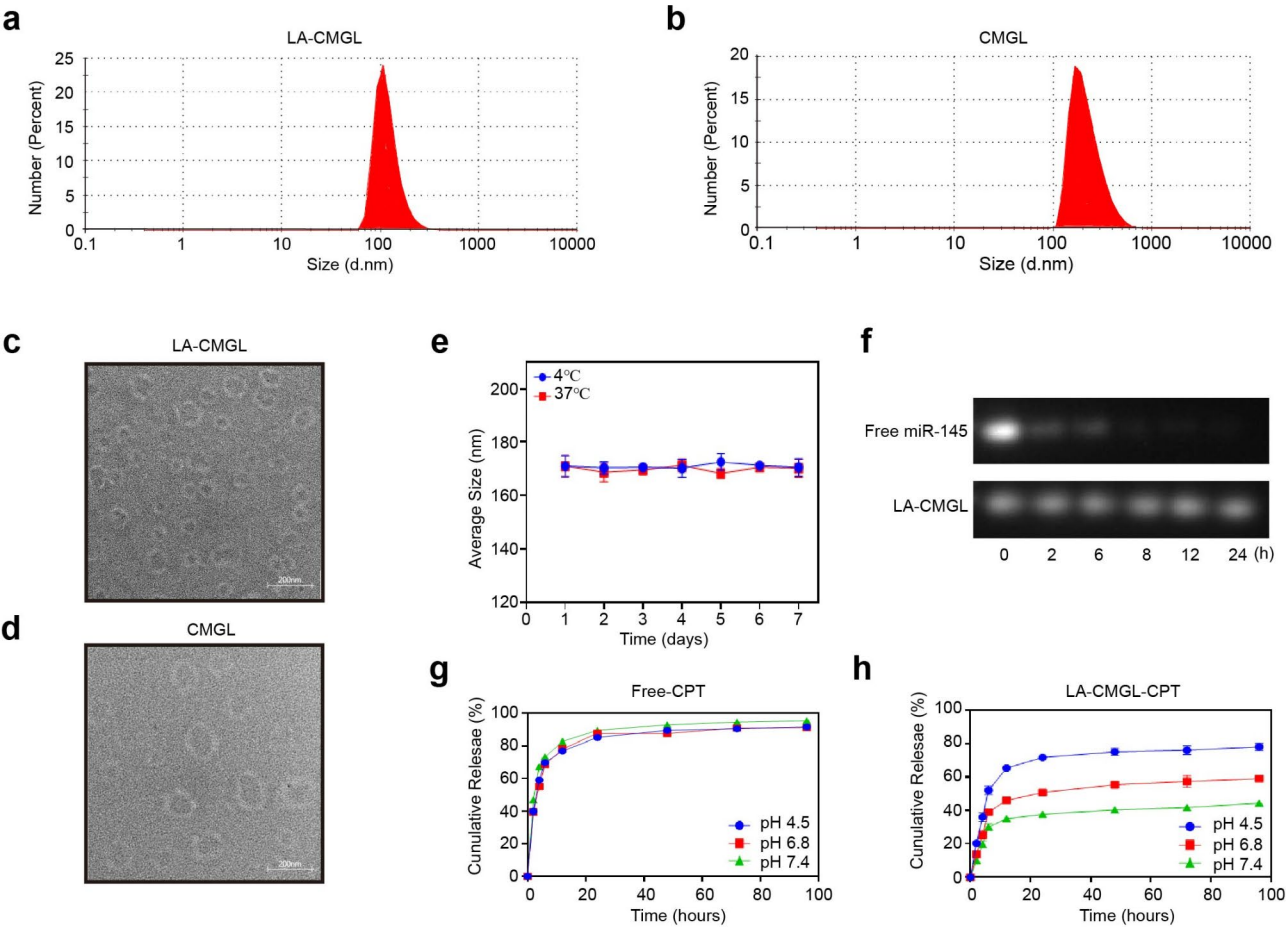
Characterizations of LA-CMGL. (**a**, **b**) The LA-CMGL and CMGL showed 160–170 nm average particle sizes, and similar morphology (**c**, **d**). (**e**) The stability of LA-CMGL during 7 days incubation with 10% FBS at 4 ℃ and 37 ℃. (**f**) Gel retardation assay after incubation with mouse serum for 2, 6, 8, 12 and 24 h. (**g**, **h**) In vitro release profile of free CPT and CPT in LA-CMGL at pH 4.5, 6.5 and 7.4. Data are mean ± standard deviation (SD)

To improve the liver-targeting ability of LNPs, DSPE-PEG-LA was generated by conjugating LA onto DSPE-PEG2000-NH2 through a one-step reaction (Figure S2a). The successful synthesis of DSPE-PEG-LA was confirmed using a ^1^H NMR analysis (Figure S2b). LA-CMGL was then prepared using the thin-film evaporation ethanol dilution method. The size, morphology, and zeta potential of LA-CMGL and CMGL are given in Fig. 1a–d and Table S1. The results showed that the LA-CMGL and CMGL were about 160–170 nm in size on average, with low PDI < 0.36, an analogous spherical shape, and compact structure. LA-CMGL also exhibited a negative surface charge with a zeta potential of -3.5 mV, enhancing the stability of LA-CMGL *via* electrostatic repulsion [40]. Next, we investigated the stability of LA-CMGL and found that LA-CMGL remained stable in the 10% FBS condition at 4 °C and 37 °C for seven days (Fig. 1e), indicating the good stability of the LNPs. We then used a gel retardation assay to evaluate the protective ability of LA-CMGL against miR-145. The results showed that LA-CMGL could protect miR-145 from degradation in mouse serum within 24 h, whereas free miR-145 was almost fully degraded within 6 h (Fig. 1f). Thus, it was inferred that LA-CMGL has a superb ability to protect miR-145 from degradation by serum nucleases, which is beneficial for prolonged blood circulation in vivo.

The encapsulation rates (EE%) of CPT and miR-145 were measured through fluorescence spectrophotometry. As shown in Table S1, LA-CMGL demonstrated high EE% for both CPT and miR-145, at about 85% and 81%, respectively. The Gd^3+^ content in LA-CMGL was determined to be 2.6 wt%. In our previous work, Gd^3+^ content of 1.8 ~ 2.64 wt% was shown to result in a favorable MR enhancement effect [5, 34]. We hypothesized that high miR-145 and CPT encapsulation rates and a moderate level of Gd^3+^ content would lead to effective synergetic antitumor properties and superb MRI efficacy. The drug release behavior of CPT in LA-CMGL was then assessed under different conditions, including a normal physiological environment (pH 7.4), an acidic tumor environment (pH 6.5), and a lysosomal environment (pH 4.5). As presented in Fig. 1g, a burst release of free CPT was observed independent of the pH value, with a 60% CPT release in the first 4 h. The release rate of CPT in LA-CMGL was faster at pH 4.5 than at pH 6.5 and pH 7.4 within the first 4 h, indicating that CPT is rapidly released from LA-CMGL in lysosomal environments. The amount of CPT released in LA-CMGL was approximately 80% at pH 4.5 within 96 h, whereas it was less than 50% and 40% at pH 6.5 and pH 7.4, respectively (Fig. 1h). Thus, we concluded that the pH-responsive drug release pattern of CPT contributes to the effective delivery of LA-CMGL to HCC cells, which is essential for further therapy.

### Targeted delivery of LA-CMGL in vitro

**Fig. 2:**
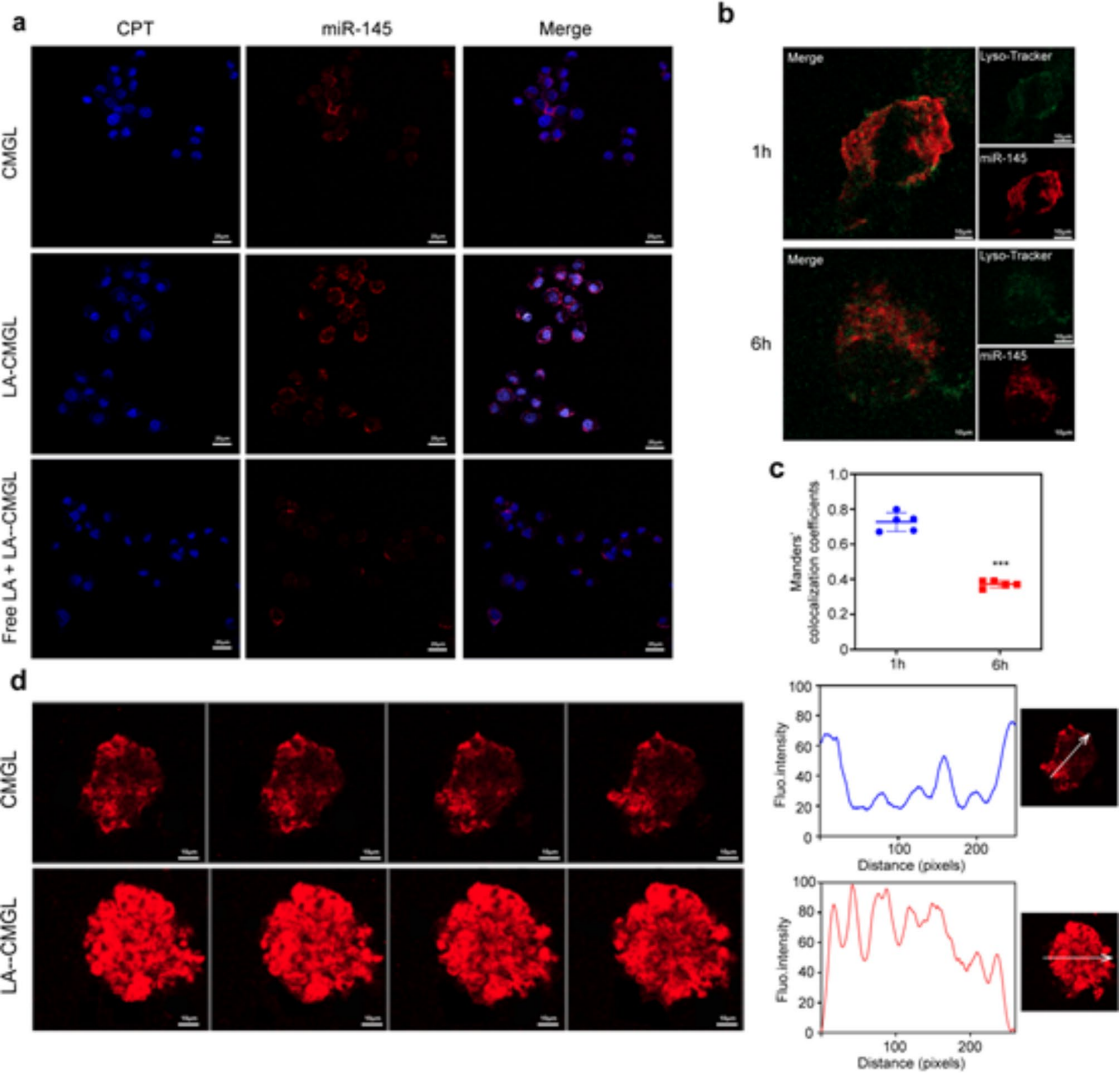
Targeting delivery and penetration ability of LA-CMGL in vitro. (**a**) Targeting delivery of CPT and miR-145 in HepG2 cells observed by CLSM (scale bar = 20 μm). (**b**) The escape ability of LA-CMGL from lysosomes in HepG2 cells at 1 h and 6 h evaluated by CLSM (scale bar = 10 μm). (**c**) Co-localization analysis of miR-145 in lysosome and cytoplasm in HepG2 cells. (**d**) Penetration of LA-CMGL (red) in HepG2 MCSs imaged by CLSM Z-stack scanning after 6 h. The MCSs surface was settled at 0 μm. Scale bar = 10 μm

Confocal laser scanning microscopy (CLSM) was conducted to assess the ability of LA-CMGL to target HepG2 cells; miR-145 was labeled with Cy5.5 (red), while CPT could emit blue fluorescence by itself. As shown in Fig. 2a, the ASGPR-overexpressed HepG2 cells treated with LA-CMGL showed stronger red and blue fluorescence than those treated with CMGL, and this effect could be inhibited by preincubating the cells with extra free LA. Notably, the blue fluorescence found in the nucleus and the red fluorescence in the cytoplasm were not affected by each other in the cells treated with LA-CMGL, indicating the successful release of CPT and miR-145 from LA-CMGL. The accumulation of CPT in the nucleus was necessary for these small chemotherapy drugs to bind with DNA to show anticancer activity [3, 18]. Additionally, a quantitative analysis involving flow cytometry (FCM) showed that LA-CMGL outperformed its nontargeted counterpart CMGL in delivering miR-145 to HepG2 cells. However, compared to CMGL, LA-CMGL did not increase the uptake of miR-145 in HepaRG cells (Figure S3). These results suggest that the modification of the LA ligand promoted cellular uptake through LA-receptor-mediated endocytosis, thus facilitating the efficient and simultaneous targeted delivery of CPT and miR-145 to HCC cells.

To further investigate the uptake process of LA-CMGL by HepG2 cells, CLSM was used to observe the escape process of LA-CMGL from lysosomes. We performed the colocalization analysis of Cy5.5-miR-145 in lysosome and cytoplasm using Manders’ colocalization coefficients. As illustrated in Fig. 2b-c, upon co-incubation with LA-CMGL for 1 h, substantial cellular uptake into HepG2 cells and a higher colocalization ratio (73.4%) between miR-145 and lysosomes was observed, suggesting 73.4% of miR-145 is located in lysosomes and the remaining is in the cytoplasm. Extending the incubation time to 6 h, the colocalization ratio decreased to (33.3%), indicating the successful lysosome escape of miR-145 (66.7% of miR-145 is located in the cytoplasm). It is possible that the mechanism for this process involved the ionizable cationic lipid DLin-MC3-DMA acquiring a charge in the acidified lysosomes, thereby promoting lysosome destabilization and cargo release into the cytoplasm [26, 27, 40]. This critical lysosome-escaping ability of LA-CMGL guaranteed the intracellular release of miR-145 in the cytoplasm and CPT in the cell nucleus, enhancing synergetic antitumor efficacy.

Next, since 3D multicellular tumor spheroids (MCSs) have been reported to recapitulate critical physiological tumor parameters in vivo and simulate various aspects of human tumor environments [32, 44], we focused on the delivery of LA-CMGL into MCSs derived from HepG2 cells. As shown in Fig. 2d, LA-CMGL penetrated entire spheroids within 6 h, which was tracked using Cy5.5 fluorescence. In contrast, CMGL was unable to penetrate the center and was limited to the cell layers outside the spheroids. These results indicate the remarkable penetration and internalization of LA-CMGL into HCC spheroids, which holds promise for inflicting synergetic cytotoxicity on HCC cells.

### Synergetic antitumor effect of LA-CMGL in vitro

**Fig. 3:**
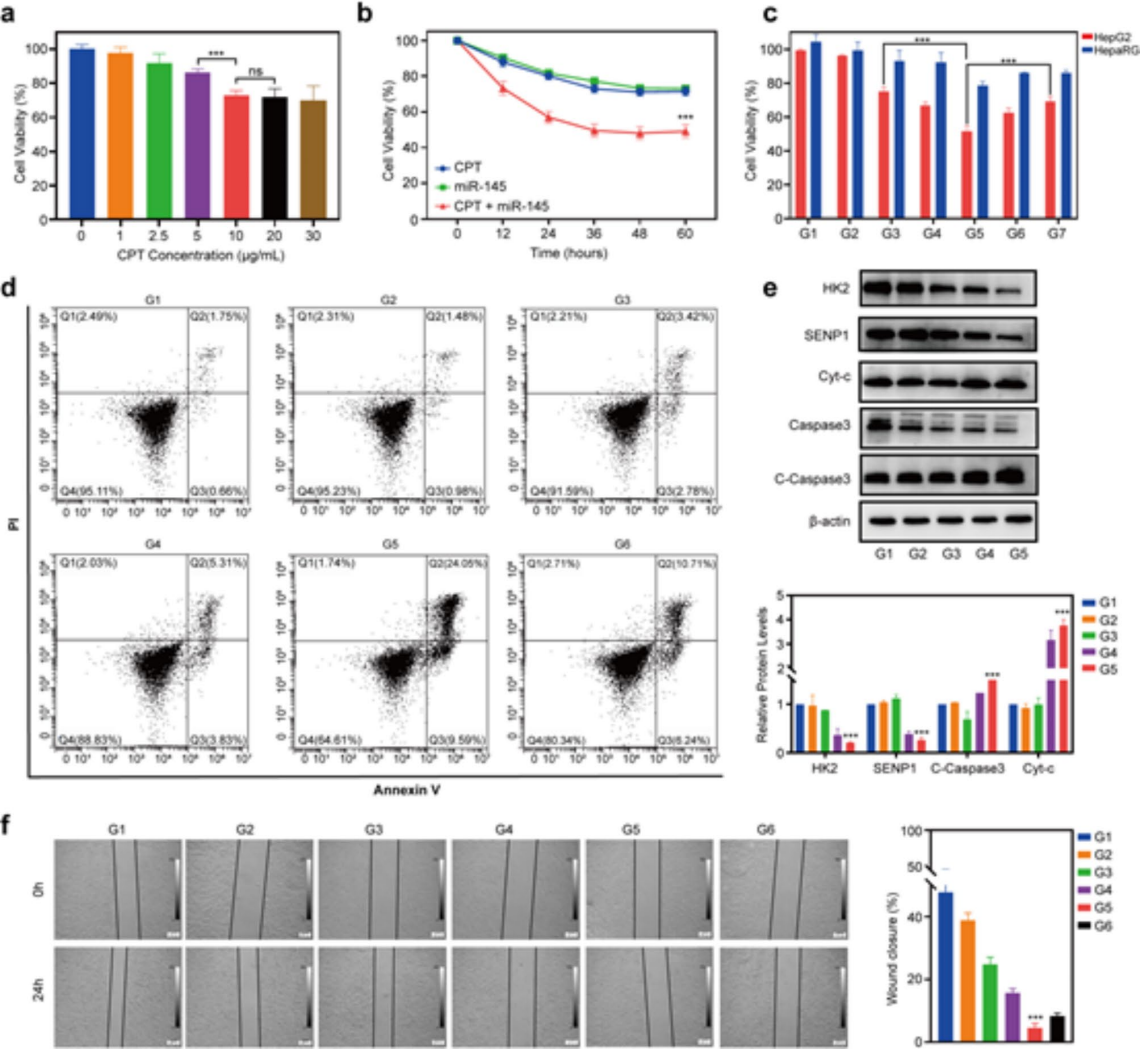
In vitro antitumor effect of LA-CMGL. (**a**) Viability of HepG2 cells treated with different dose levels of CPT for 24 h (*n* = 3). (**b**) Viability of HepG2 cells incubated with CPT, miR-145, CPT + miR-145 from 12 to 60 h (*n* = 3). (**c**) The proliferation inhibition of HepG2 cells and HepaRG cells treated with different formulations (G1-7) with the final miR-145 concentration of 100 nM and CPT concentration of 10 µg/mL. G1, G2, G3, G4, G5, G6 and G7 represented PBS, LA-NC-L, LA-CPT-L, LA-miR-145-L, LA-CMGL, CMGL and CPT + miR-145 groups, respectively (*n* = 3). (**d**) Flow cytometry analysis of the apoptosis ratios in HepG2 cells treated with different formulations (*n* = 3). (**e**) WB analysis showed LA-CMGL significantly increased the expression of C-caspase3 and Cyt-c protein. β-actin was used as an internal control. (**f**) In vitro wound scratch assay of the HepG2 cells (*n* = 3). Quantitative analysis was performed using Image J software (*n* = 3). Data are mean ± standard deviation (SD). Statistical significances in (**a**), (**c**), (**e**) and (**f**) were calculated via the Student’s *t* test (***p < 0.001). Statistical significances in (**b**) were calculated *via* the one-way ANOVA with Tukey’s *post hoc test* (***p < 0.001)

To explore the synergetic cytotoxic effects of CPT and miR-145 against HepG2 cells, a CCK8 assay was performed using free CPT and miR-145 drugs without a nanocarrier. As shown in Fig. 3a, the upregulation of CPT inhibited the survival rate of HepG2 cells in a slight dose–effect relationship. The peak inhibitory effect of CPT on HepG2 cell viability was seen at a CPT concentration of about 10 µg/mL, so this was chosen as a favorable concentration. Meanwhile, the concentration of 100 nM miR-145 was selected for miR-145 application according to previous reports [8, 23]. We found that treating the HepG2 cells with free CPT + free miR-145 resulted in cell viability rates of 57.37% and 48.25% after 24 h and 48 h of incubation, respectively, which were higher than the viability rates resulting from treatment with free CPT (79.86% and 71.53%) (Fig. 3b). In addition, CCK8 assay against Huh7 and Hep3B cells showed similar results with those of HepG2 cells (Figure S4). A Western blotting (WB) analysis showed that the levels of protein SENP1 and HK2 decreased, the apoptotic proteins cleaved-caspase3 (C-caspase3) and cytochrome-c (Cyt-c) increased in the HepG2, Huh7 and Hep3B cells after treatment with CPT + free miR-145, compared to treatment with CPT alone (Figure S5). These data confirmed clearly that the ability of miR-145 to significantly enhance the chemotherapeutic efficacy of CPT.

We then performed a CCK8 assay to investigate whether the co-delivery of CPT and miR-145 by LNPs provides better results. As shown in Fig. 3c, the inhibitory effects of the LA-miR-145 LNPs (abbreviated as LA-miR-145-L) and LA-CPT LNPs (LA-CPT-L) on the viability of HepG2 cells were comparable to that of free CPT + miR-145 (*p* > 0.05). Most importantly, a striking decrease in HepG2 cell viability was observed with the use of LA-CMGL (55.31% viability), compared to the use of LA-CPT-L (72.34%), LA-miR-145-L (64.79%), or CMGL (60.26%) (all *p* < 0.01). Additionally, the survival rate of HepG2 cells treated with blank LA LNPs (LA-NC-L) exhibited no significant difference from the control groups (*p* > 0.05), indicating the biocompatibility of the drug carrier. Next, we used FCM to compare the apoptosis ratios of HepG2 treated with different LNP formulations (Fig. 3d). Our data revealed that HepG2 cells treated with LA-CMGL exhibited a significantly higher apoptosis ratio (33.64%) than those treated with LA-CPT-L (6.2%), LA-miR-145-L (9.14%), or CMGL (16.95%) (all *p* < 0.01), which correlated well with the CCK8 assay results. Moreover, a WB analysis confirmed that, compared to LA-CPT-L and LA-miR-145-L, LA-CMGL significantly increased the levels of C-caspase3 and cyt-c in the HepG2 cells (Fig. 3e).

Next, to investigate anti-cell migration activity of LA-CMGL against HepG2, the wound scratch assay was performed. As shown in Fig. 3f, all drug formulations presented obviously inhibitory effect in comparison with the control and LA-NC-L groups after 24 h. The CMGL groups showed slightly higher inhibitory effect than LA-CPT-L and LA-miR-145-L groups, while LA-CMGL groups exhibited obviously stronger inhibit migration activity. The wound closure rate of LA-CMGL, LA-CPT-L and LA-miR-145-L was 4.6%, 22.3% and 14.7%, respectively. Similar trends were also observed in Huh7 and Hep3B cells (Figure S6). Taken together, these results suggest that LA-CMGL-mediated co-delivery of miR-145 and CPT is more effective than LA-CPT-L or LA-miR-145-L treatment, as it enhances the antiproliferation effect, cell apoptosis and anti-cell migration activity.

### Synergetic antitumor mechanism of CPT and miR-145

**Fig. 4:**
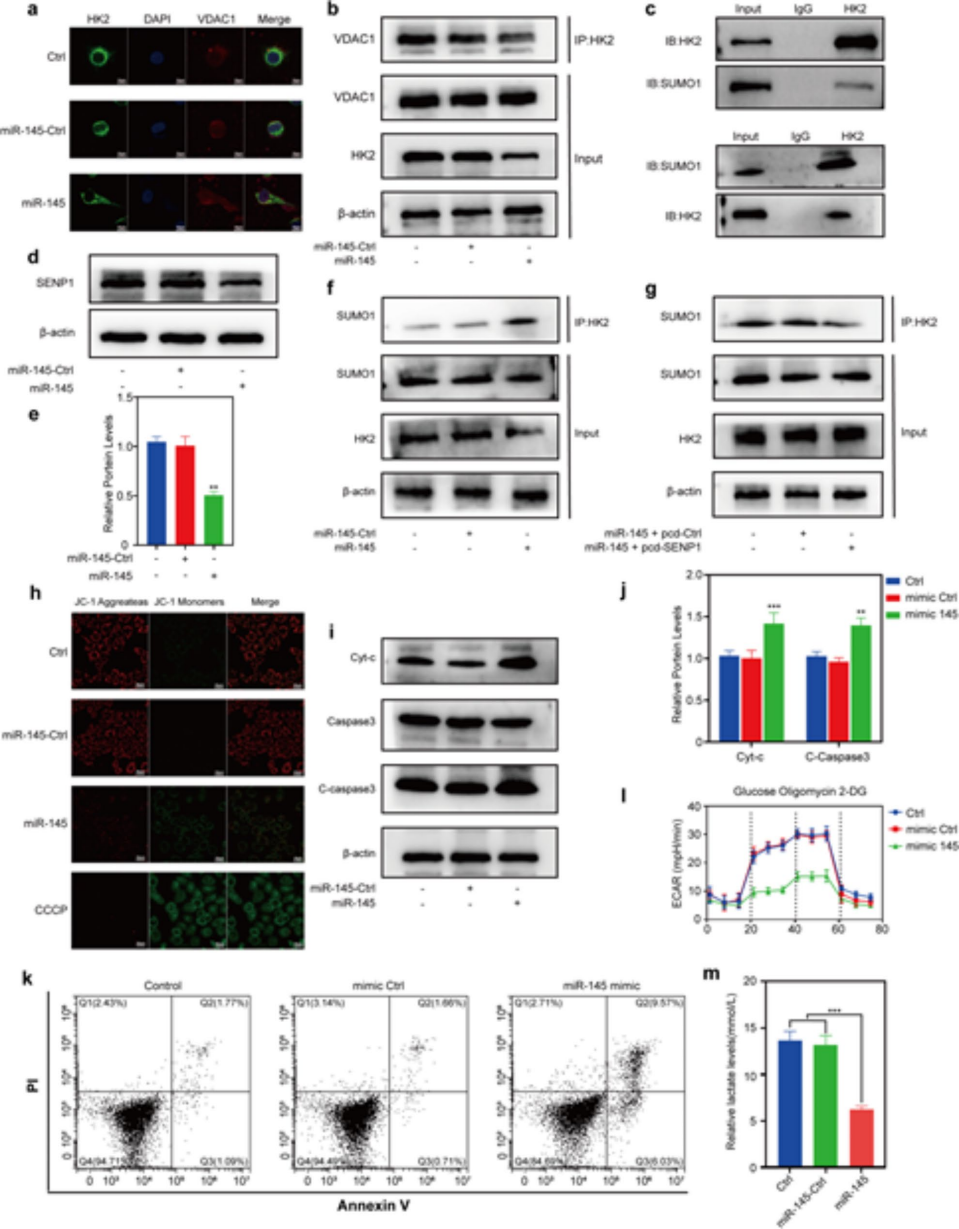
Synergetic antitumor mechanism of CPT and miR-145. (**a**) Immunofluorescence staining of HK2 (green) and VDAC1 (red) co-localization after HepG2 transfection with miR-145. (**b**) Co-IP detection of HK2 binding to VDAC1 after miR-145 transfection. (**c**) Immunoprecipitation of HK2 and SUMO1. (**d**) Expression and (**e**) quantification of SENP1 protein and after miR-145 transfection. (**f**) Co-IP of HK2 with SUMO1 after miR-145 transfection. (**g**) Co-IP detection SUMOylation of HK2 after cotransfection. (**h**) Detection of mitochondrial membrane potential after miR-145 transfection. (**i**) Protein expression and (**j**) quantification of Cyt-c, C-Caspase3 and Caspase3 after miR-145 transfection. (**k**) Detection of apoptosis after miR-145 transfection by flow cytometry. (**l**) HepG2 extracellular acidification rate after miR-145 transfection. (**m**) Lactate content of medium after miR-145 transfection or co-transfection. Statistical significance in (**e**), (**j**) and (**m**) was calculated *via* the Student’s *t* test (**p < 0.01)

Next, we explored the mechanisms underlying the synergetic cytotoxic effects of CPT and miR-145 in LA-CMGL. As a promising antitumor drug target, HK2 has dual regulatory effects on the metabolic and proliferative activities of cancer cells [45, 46]. HK2 binds to voltage-dependent anion-selective channel protein 1 (VDAC1) on the mitochondrial surface, which contributes to the inhibition of apoptosis by closing the mitochondrial permeability transition pores and preventing cyt-c release [46]. We found that miR-145 mimics significantly decreased the colocation of HK2 and VDAC1 in HepG2 cells (Fig. 4a). Moreover, the GPS-SUMO database (Figure S7) and co-inmunoprecipitation (Co-IP) assays (Fig. 4b) showed that HK2 could be modified by SUMOylation (Fig. 4c). Since SUMOylation (a reversible posttranslational modification) reportedly disrupts the binding of HK2 to VDAC1 [47, 48], we attempted to explore whether miR-145 would alter the binding of HK2 to VDAC1 by affecting HK2 SUMOylation. SENP1, an important de-sumo protein, is largely responsible for the deconjugation of SUMO1 modifications [42, 48]. As show in Figure S8, the expression of SENP1 was markedly increased in HCC tumors and cells (HepG2, Huh7, and Hep3B). Altering SENP1 expression significantly affected the SUMOylation level of HK2 and it’s binding to VDAC1 (Figure S9, S10). Now, the results showed that miR-145 inhibitor promoted the expression of SENP1 (Figure S12c), while miR-145 mimics inhibited the expression of SENP1 (Fig. 4d-e). Moreover, both ENCORI database predictions (Figure S11) and Dual-Luciferase Reporter results (Figure S12a) showed that SENP1 was one of the targets regulated by miR-145. Additionally, the Co-IP results showed that miR-145 robustly increased the SUMOylated HK2 levels (Fig. 4f), whereas co-transfection miR-145 mimics and SENP1 plasmid decreased the SUMOylated HK2 levels (Fig. 4g). Taken together, these results indicate that SENP1 functions as the key HK2 deSUMOylase.

We then evaluated how miR-145 promotes the apoptosis of HepG2 cells. As expected, miR-145 decreased the binding of HK2 to VDAC1 and the mitochondrial membrane potential (Fig. 4h). In addition, miR-145 increased the expression of C-caspase3 and cyt-c (Fig. 4i-j) and the apoptosis rate of HepG2 cells (Fig. 4k) which were accompanied by decreased levels of ECAR (Fig. 4l) and extracellular lactate (Fig. 4m). The simultaneous overexpression of miR-145 and SENP1 reversed the above effects of miR-145 (Figure S13a–d). Thus, the above results strongly suggest that miR-145 promotes the apoptosis of HepG2 cells and then sensitize cancer cells to CPT by targeting the SENP1-mediated HK2 SUMOylation and glycolysis pathways.

### Tumor inhibitory efficacy and safety evaluations in vivo

To evaluate the antitumor effect of LA-CMGL in an actual liver environment, a DEN + CCl_4_-induced HCC mouse model was established, and hepatocellular tumors were initiated and promoted *via* a two-stage application of DEN and CCl_4_ [43]. This model is relevant for understanding human HCCs associated with chronic liver injury, inflammation, and fibrosis/cirrhosis [43, 49], so we utilized it in the present study to simulate a difficult delivery challenge with a late-stage disease. This model was first used to evaluate inhibitory efficacy, followed by systemic toxicity.

**Fig. 5:**
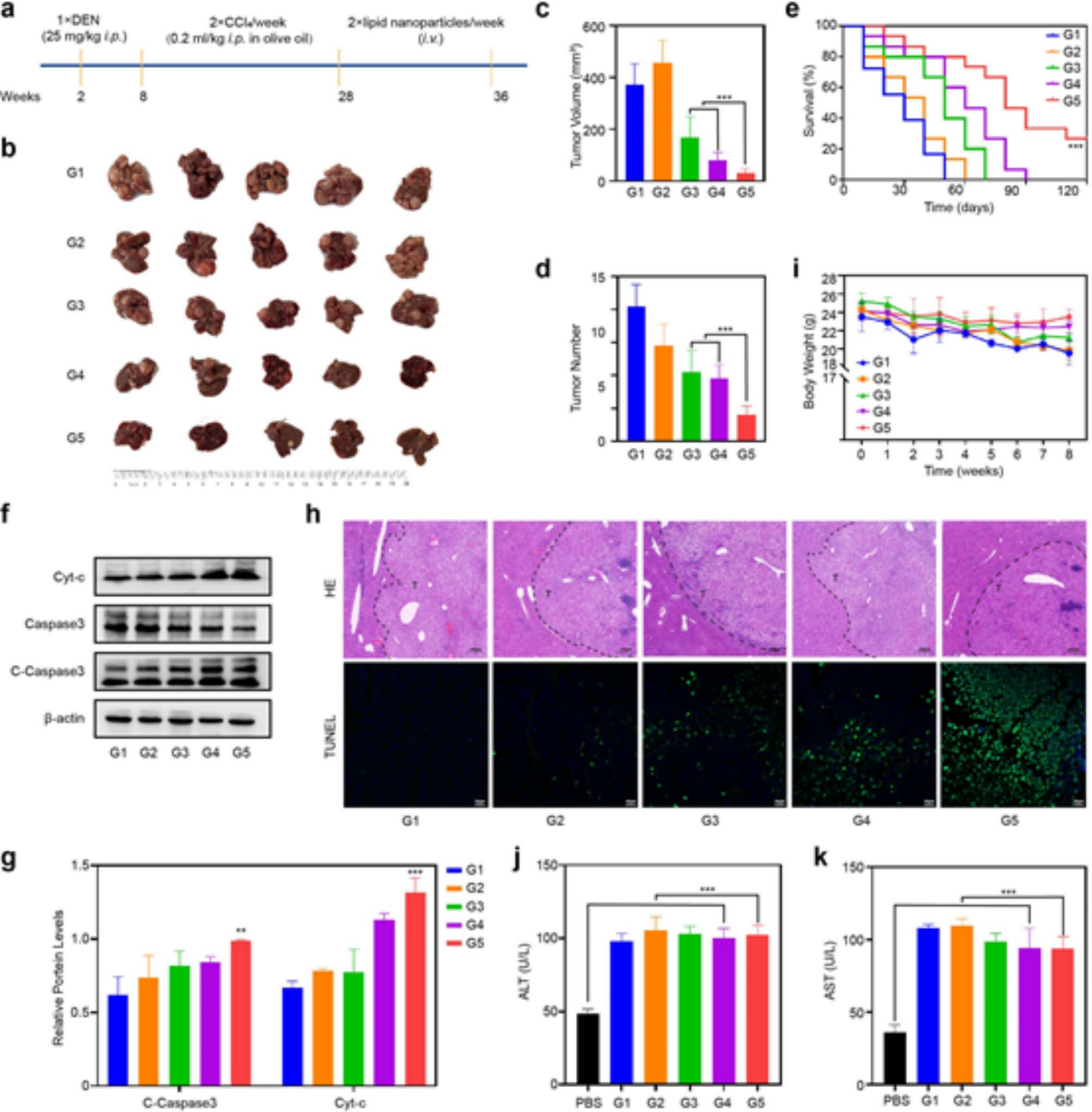
In vivo antitumor efficacy and safety evaluation. (**a**) Schemetic illustration of DEN + CCl_4_-induced HCC mouse model and administration regimen for systemic therapy. (**b**) The representative gross images of HCC tumor masses in different groups at 36th week after treatment (*n* = 5). (**c**, **d**) Largest tumor volume and the number of tumor masses of model mice treated with different formulations (*n* = 5). (**e**) Survival curves of model mice after different treatments (*n* = 7). (**f**) The WB results showed LA-CMGL significantly increased the expression of C-Caspase3 and cyt-c protein in tumor tissue. (**g**) Quantification of WB by Image J software (*n* = 3). (**h**) H&E and TUNEL staining of tumor sections (*n* = 5) after different treatments. T indicate tumor tissue. Scale bars are 200 μm. (**i**) Weight changes of model mice after different treatments (*n* = 5). (**j**, **k**) Serum ALT and AST levels in model mice (*n* = 5). Healthy mice treated with PBS were used as control (*n* = 3). Data are mean ± standard deviation (SD). Statistical significances in (**e**), and (**i**) were calculated *via* the one-way ANOVA with Tukey’s *post hoc test* (***p < 0.001). Statistical significance in (**c**), (**d**), (**g**), (**j**) and (**k**) was calculated *via* the Student’s *t* test (**p < 0.01, ***p < 0.001)

We initiated a therapeutic regimen from week 28 after introducing HCC into the mouse model, which involved the administration of different LNP formulations containing 10 µg/mL CPT and/or 100 nmol/L miR-145 twice a week (Fig. 5a). In the 36th week, ex vivo liver imaging (Fig. 5b) confirmed that the mice that received LA-CMGL treatment had minimal tumor volumes and tumor numbers compared to the single drug-loaded groups (Fig. 5c-d). More importantly, LA-CMGL significantly prolonged the survival time of tumor-bearing mice to 121 days, whereas the other LNPs were not able to extend survival past 90 days (Fig. 5e). A WB analysis showed that the expression levels of the apoptosis-related proteins C-caspase3 and cyt-c were significantly higher in the LA-CMGL group than in the other groups (Fig. 5f). Hematoxylin and eosin (H&E) or terminal deoxynucleotidyl transferase dUTP nick end labeling (TUNEL) staining further confirmed these results (Fig. 5g). Thus, LA modification played a crucial role in the targeted co-delivery of CPT and miR-145, resulting in synergetic tumor inhibition and improved therapeutic effects.

We subsequently estimated the systemic toxicity levels of different LNPs formulations. As depicted in Fig. 5h, the body weights of all the LNP-treated mice kept growing slowly or remained unchanged, indicating that negligible systemic side effects were generated during the co-delivery of CPT and miR-145 by LA-CMGL. Moreover, no histopathological alterations were observed in the major organs of the LA-CMGL-treated mice (Figure S14). We also performed a blood study to evaluate the potential clinical translation of the LA-CMGL. Due to liver injury induced by DEN + CCl_4_, ALT and AST levels increased sharply in the HCC model mice, as compared to the healthy mice, and LA-CMGL treatment slightly decreased these levels (Fig. 5i, j). There were no obvious differences between the PBS- and LA-CMGL-treated mice with respect to the liver damage induced by DEN + CCl_4_. These findings highlight the excellent biocompatibility and biosafety of LA-CMGL.

### In vitro and in vivo MRI analyses

**Fig. 6:**
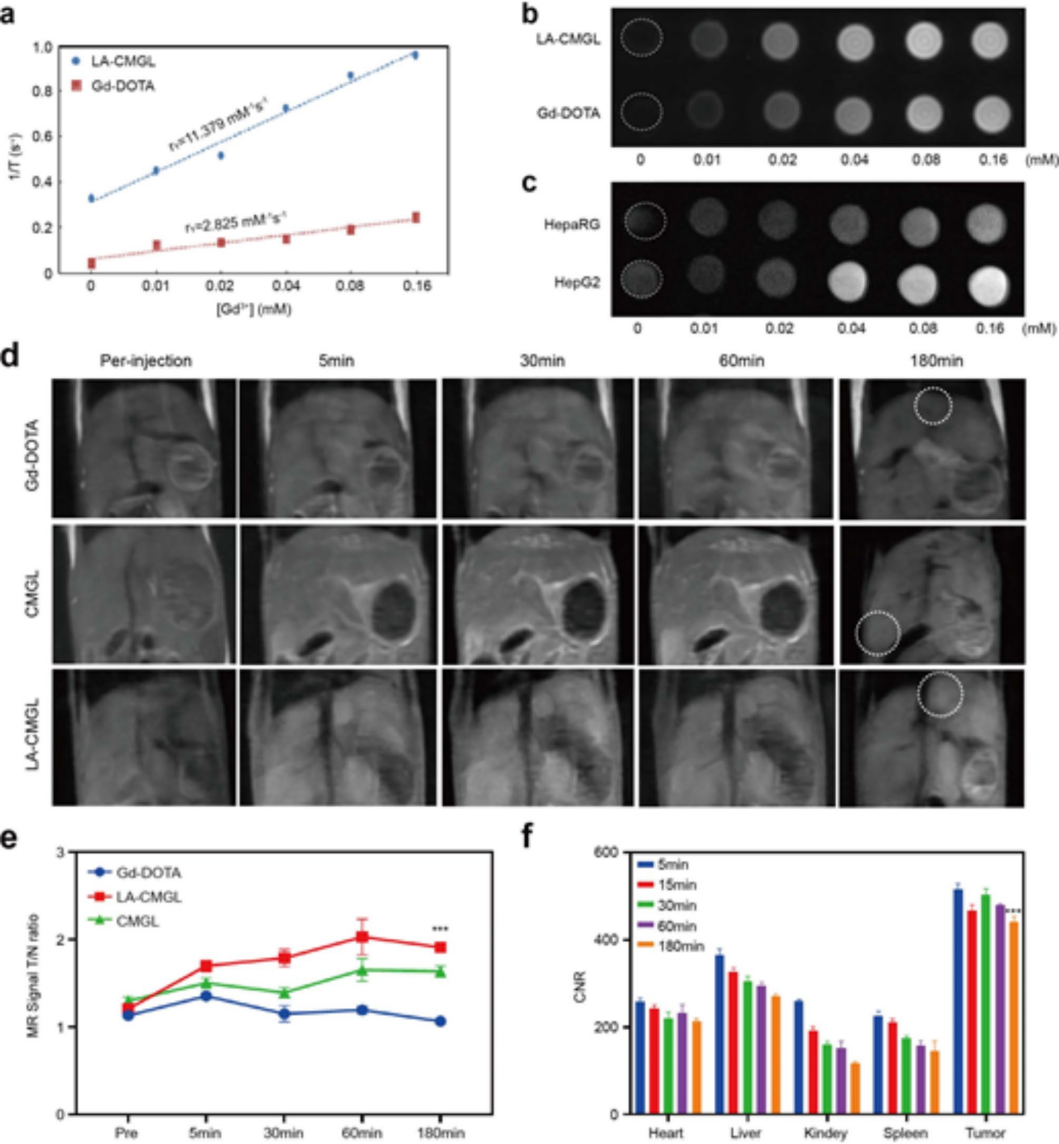
MR imaging relaxivity and in vivo MR imaging of LA-CMGL. (**a**) Longitudinal relaxation rate (1/T1) of Gd-DOTA and LA-CMGL in an aqueous solution as a function of the Gd^3+^ concentration. (**b**) T1-weighted MR images of Gd-DOTA and LA-CMGL at different Gd^3+^ concentrations. (**c**) T1-weighted MR images of HepG2 and HepaRG cells incubated with LA-CMGL at different concentrations. (**d**) T1-weighted MR enhancement of HCC model mice at various time points following the intravenous injection of Gd-DOTA, CMGL, and LA-CMGL. (**e**) Tumor and normal ratio (T/N) for the liver of HCC mice at pre-injection, 5, 30, 60 and 180 min after intravenous injection of the solution of LA-CMGL (*n* = 3). (**f**) Contrast-to-noise ratio (CNR) of the heart, liver, kidney, spleen, and tumor in the HCC mice before injection and at 5, 15, 30, 60 and 180 min after intravenous injection of the solution of LA-CMGL (*n* = 3). Data are mean ± standard deviation (SD). Statistical significances in (**e**) were calculated *via* the one-way ANOVA with Tukey’s *post hoc test* (***p < 0.001). Statistical significance in (**f**) was calculated *via* the Student’s *t* test (**p < 0.01)

To investigate the possibility of using LA-CMGL as a T1-weighted MRI contrast agent, the relaxivities (r1) of LA-CMGL and Gd-DOTA were evaluated. The r1 value for LA-CMGL was 11.379 mM^− 1^S^− 1^, which was nearly four times the 2.825 mM^− 1^S^− 1^ of Gd-DOTA (Fig. 6a). Furthermore, in a 1.5T MRI, LA-CMGL exhibited much better signal contrast and brightness than Gd-DOTA with the same Gd^3+^ concentration (Fig. 6b). This may be attributed to an increase in the local concentration of Gd^3+^ and a decrease in the rate of molecular tumbling [32, 33]. In addition, as the concentration of Gd^3+^ gradually increased, the T1 signal improved in both HepG2 and HepaRG cell lines. Compared to the HepaRG cells, the HepG2 cells exhibited a much higher signal under the same Gd^3+^ conditions (Fig. 6c). This result strongly confirmed the site-specific accumulation and superior intracellular MRI contrasting effect of LA-CMGL due to ASGPR-mediated endocytosis [5].

Based on the preceding cellular MRI results, we further investigated the tumor-targeting properties and biodistribution of LA-CMGL in vivo. As shown in Fig. 6d, Gd-DOTA was almost nonexistent in the cancerous tissue 180 min after injection, but LA-CMGL were found in local tumors. This prolonged enhancement of LA-CMGL in tumor environments would be beneficial for constant drug release. In addition, after injecting Gd-DOTA for 5 min, the enhancement of MRI signals could be identified. However, there was little distinction between the MRI signals for the tumor tissue and the adjacent normal liver tissue, and the tumor boundary was blurred. This can be explained by extracellular imaging property of Gd-DOTA rather than specific intracellular accumulation. In contrast, the tumor boundary was distinct after LA-CMGL injection compared with those of Gd-DOTA and CMGL at 5 min and particularly apparent at 30 min. This may be attributed to the targeted delivery of LA-CMGL into tumor cells/tissues, resulting in intracellular imaging.

For quantitative analysis, the tumor-to-normal ratio (T/N) of LA-CMGL was calculated, where T and N denote the signal intensities of the different Gd^3+^ preparations in the region of tumor and the normal brain tissue, respectively. As depicted in Fig. 6e, LA-CMGL was stronger than those of Gd-DOTA and CMGL (all *p* < 0.05) 15 min post injection, allowing for HCC to be identified at an early stage based on the clear boundary and shape of the tumor. Furthermore, to monitor the distribution of LA-CMGL in tumor-bearing mice, contrast-to-noise ratio (CNR) measurements were taken. As anticipated, LA-CMGL had a higher CNR in the liver than in the other organs (Fig. 6f). This result aligned with the in vivo and ex vivo fluorescence imaging findings (Figure S15), indicating that the LA-CMGL composed of four components in a fixed ratio were optimal for liver delivery, despite the introduction of CPT/miR-145 and Gd-DOTA. Most importantly, LA-CMGL had a higher CNR in cancerous tissues than that in other organs at any time point (*p* < 0.01), demonstrating that the LA-CMGL possessed good tumor-targeting accumulation abilities; this finding is consistent with the reports of previous cellular studies. Considering that Gd^3+^, CPT, and miR-145 were coloaded in LA-CMGL, it is likely that the MRI signal intensity of LA-CMGL in the major organs reflected the concentrations of the drugs distributed in these organs. Therefore, it is reasonable to assume that this quantitative analysis would be an efficient, real-time approach for noninvasively monitoring drug distribution. In sum, these results clearly show the strengths of using LA-CMGL for HCC target imaging and the real-time visualization of drug delivery and biodistribution in vivo, which is of great importance for tumor monitoring and treatment guidance.

## Conclusion

In this study, we designed LA-modified LNPs (LA-CMGL) with coloaded CPT/miR-145 and Gd-DOTA for simultaneous targeted therapy and MRI contrast enhancement for HCC. The results showed that the LA modification enabled LA-CMGL to precisely deliver CPT/miR-145 into tumor cells and tissues. In vitro and in vivo antitumor analyses demonstrated that the LA-CMGL were more effective than free drugs or single drug-loaded LNPs. Mechanistically, miR-145 may sensitize cancer cells to CPT and promote apoptosis by targeting SENP1-mediated HK2 SUMOylation and glycolysis pathways. Moreover, in vitro and in vivo tests confirmed that the loaded Gd-DOTA could serve as an effective T1-weighted contrast agent for tumor detection and drug delivery monitoring. In sum, the LA-modified chemo-gene co-delivery system developed in this work shows great potential as a theranostic system for personalized cancer therapy.

### Electronic supplementary material

Below is the link to the electronic supplementary material.

Supplementary Material 1

## Data Availability

The data of this study is available from the corresponding authors on reasonable request.

## Abbreviations

CPT: Camptothecin
HCC: Hepatocellular carcinoma
LA: Lactobionic acid
LNPs: Lipid nanoparticles
DEN: Diethylnitrosamine
SENP1: SUMO-specific peptidase 1
HK2: Hexokinase
miRNA: MicroRNA
ECAR: Extracellular acidification rate
MC3: DLin-MC3-DMA
ASGPR: Asialoglycoprotein receptors
DLS: Dynamic light scattering
TEM: Transmission electron microscopy
EE%: Encapsulation efficiencies
ICP: OES-Inductively coupled plasma-optical emission spectrometer
PDIs: Polydispersity indexes
MWCO: Molecular weight cut off
CLSM: Confocal laser scanning microscopy
CCK8: Cell Counting Kit-8
WB: Western blotting
CNR: Contrast-to-noise ratio
ROI: Region of interest
MCSs: Multicellular tumor spheroids
C: caspase3-Cleaved-caspase3
Cyt: c-Cytochrome-c
VDAC1: Voltage-dependent anion-selective channel protein 1
Co: IP-Co-inmunoprecipitation
H&E: Hematoxylin and eosin
TUNEL: Terminal deoxynucleotidyl transferase dUTP nick end labeling
ALT: Alanine transaminase
AST: Aspartate aminotransferase
