# Supplementary material for: Co-delivery of camptothecin and MiR-145 by lipid nanoparticles for MRI-visible targeted therapy of hepatocellular carcinoma
Source: J Exp Clin Cancer Res. 2024 Aug 30;43:247. doi: 10.1186/s13046-024-03167-9 (PMC11363558; doi:10.1186/s13046-024-03167-9)

Supporting Information

Co-Delivery of Camptothecin and MiR-145 by Lipid Nanoparticles for MRI-Visible Targeted Therapy of Hepatocellular Carcinoma

Jing Rong^1†^, Tongtong Liu^2†^, Xiujuan Yin^1^, Min Shao^1^, Kun Zhu^1^, Bin Li^1^, Shiqi Wang^2^, Yujie Zhu^1^, Saisai Zhang^1^, Likang Yin^1^, Qi Liu^2*^, Xiao Wang^1*^, and Lei Zhang^2*^

^1^ Department of Radiology, the First Affiliated Hospital of Anhui Medical University, Anhui Medical University, Hefei 230032, China

^2^ School of Pharmacy, Key Laboratory of Anti-inflammatory of Immune Medicines of Ministry of Education, Inflammation and Immune Mediated Diseases Laboratory of Anhui Province, Anhui Institute of Innovative Drugs, Anhui Medical University, Hefei 230032, China

*E-mail (Q.L.): liuqi@ ahmu.edu.cn

*E-mail (X.W.): [wangxiao@ahmu.edu.cn](mailto:wangxiao@ahmu.edu.cn)

*E-mail (Z.L.): zhanglei-1@ahmu.edu.cn

**Table of Contents**

**Materials and Methods 3-7**

**Figure S1 8**

**Figure S2 8**

**Figure S3 9**

**Figure S4 9**

**Figure S5 10**

**Figure S6 11**

**Figure S7 12**

**Figure S8 12**

**Figure S9 13**

**Figure S10 13**

**Figure S11 14**

**Figure S12 14**

**Figure S13 15**

**Figure S14 16**

**Figure S15 16**

**Table S1 17**

**Table S2 17**

**MATERIALS AND METHODS**

**Cell Culture**

HepG2 (human hepatoma cell line) and HepaRG (human liver cell line) were obtained from Beijing Bio Research Institute (China). These cells were cultured in DMEM with 10% with fetal bovine serum (FBS, 10%, v/v) and 1% penicillin/

streptomycin, respectively, in a cell incubator (37 °C, 5% CO_2_) under fully humidified conditions.

**Multicellular Spheroids (MCs) Preparation**

The HepG2 multicellular tumor spheroid (MCS) were established according previous studies. ^1^ Briefly, the HepG2 single-cell suspension (5,000 cells) was transferred into a 48-cell culture plates precoated with 2% (w/v) sterilized agarose. Then, the HepG2 MCSs were allowed to culture in the medium grow for 1 week (37 °C, 5% CO_2_). When the tumor spheroids formed, LA-CMGL and CMGL in DMEM (1 mg/mL, pH 7.0) were added into the plates. After 6 h, the MCs were harvested, washed with PBS (pH 7.4, 0.01 M) thrice, and imaged by CLSM.

**Animal Models and Human Samples**

Male C57BL/6 mice (12 days of age, ~12 g) were purchased from the animal center of the Cancer Institute of Chinese Academy of Medical Science, and bred at Animal Experimental Center, Anhui Medical University. All experimental protocols were conducted within Anhui Medical University guidelines for animal research and were approved by the Animal Experiment Ethics Review of Anhui Medical University (approval no. LLSC20221110). The DEN + CCl_4_ Induced HCC mice model was established according to previous report. ^2^ Male C57BL/6 mice were administered intraperitoneal (*i.p.*) injection of a single dose of 25 mg/kg N-nitrosodiethylamine (DEN) at 14 days of age. Then, CCl_4_ 0.2 ml/kg, 10% dissolved in olive oil was administered *i.p.* 2 times per week starting at 8 weeks of age for up to 28 weeks, at which point to induce HCC 100% model as described previously.

Liver tumor tissues and non-tumor tissues came from patients suffered HCC undergoing resection via the tissue procurement facility at the First Affiliated Hospital of Anhui Medical University. Medicine's Ethics Committee of Anhui Medical University approved this study (approval no. 2023-467). Sample collection was conducted under supervision of the ethics review board and all patients, or their guardians provided written informed.

**Selective Organ targeting (SORT) Property of LA-CMGL**

To explore the selective liver-targeting property of LA-CMGL, we administered saline, free cy5.5-miR-145 and LA-CMGL (at miR-145 concentration of 100 nmol/L) *i.v.* into the normal C57BL/6 mice. At 6 h, mice were euthanized and whole body images were acquired using an *in vivo* IVIS Lumina Imaging technique (Caliper Life Sciences). Then the mice were killed and major organs were removed (heart, liver, spleen, lung and kidney) and detected *ex vivo*.

**Western Blotting**

HepG2 cells were seeded into 6-well plates and treated with PBS, LNP-NC-L, LA-CPT-L, LA-miR-145-L, CMGL and LA-CMGL for 24 h. Then, the cells were collected and proteins were extracted according to the recommended protocols. Proteins were separated using 12% SDS-PAGE gel and transferred to PVDF membranes (Millipore, Billerica, MA, USA), which were blocked in 5% skim milk for 1 h and then using primary antibodies and secondary antibodies conjugated with HRP. For immunoblotting, the following antibodies were used: anti-SENP1 (Proteintech, 25349-1-AP, 1:1000), anti HK2 (Proteintech, 22029-1-AP, 1:1000), anti-SUMO1 (Santa,sc-5308,1:1000), anti-VDAC1 (Proteintech,66345-1-lg, 1:1000), anti-Caspase3 (Wanleibio,WL04004, 1:1000), anti-Cleaved-caspase 3 (Wanleibio,WL02117, 1:1000), anti-Cytc (Wanleibio,WL02410, 1:1000), anti-β-actin (ZSGB-BIO, TA-09, 1:1000). The detection method of protein in tissue is the same as above.

**RNA Isolation and Real-Time Quantitative PCR**

Total RNA was extracted from tissues or HepG2 cells using TRIzol reagent (AG21102, Accurate Biology, China) The obtained Total RNA was reverse transcribed into cDNA using 5 × Evo M-MLV RT Master Mix kits (AG11706, Accurate Biology ,China). qRT‐PCR was carried out using a NovoStart SYBR qPCR SuperMix plus Kit (E096-01B, Novoprotein, China) on a CFX96 real‐time PCR system (BioRad, USA). The mRNA ratio of the target gene to β-actin was calculated by using the 2^−ΔΔCt^ formula. The experiments were performed at least three times using three different templates.

**Mitochondrial Membrane Potential**

Mitochondrial membrane potential (MMP) was assessed using JC-1 kit (Elabscience, China). JC-1 acts as a fluorescent probe that can quickly detect changes in the MMP, thereby serving as an early marker of cell apoptosis. Cells were incubated with JC-1 working solution for 20 min at 37 °C in the dark. Cells were washed with cold JC-1 staining buffer for 2 times and observed by fluorescence microscopy. Red J-aggregate emission (Ex/Em=585/590 nm) was observed in normal cells, and JC-1 monomers (Ex/Em=514/529 nm) were generated as membrane potential decreased, resulting in green emission.

**Co-IP and Immunoblot Analysis**

Tissues or cells were lysed 20~30 min in IP buffer (1.0% [vol: vol] Protease inhibitors: Cell lysis buffer). After centrifugation for 15 min at 14,000 g, supernatants were collected and incubated with IgG antibody and IP antibody respectively. After of incubation overnight, add beads, which were washed tow times with Cell lysis buffer, into two groups and incubate 60 min. Immunoprecipitates were eluted by boiling with 25% (vol: vol) 5×SDS sample buffer and boiled at 100°C for 5 min then followed by western blot. Equal amounts of extracts were separated by 10%SDS-PAGE gel, and then they were transferred onto PVDF membranes and then blotted with specific antibody. Co-Immunoprecipitation Kit (BersinBio, Bes3011, China).

**Immunofluorescence and Confocal Microscopy**

HepG2 were seeded into the uncoated 35-mm dishes. After cell transfections, cells were washed in cold PBS twice, fixed with 4% freshly prepared formaldehyde in PBS for 10-20 min, and then washed three times with PBS. Blocked in 3% bovine serum albumin for 30 min, incubated in the primary antibodies anti-HK2 (Proteintech, 22029-1-AP, dilution 1:100) or anti-VDAC1 (Proteintech,66345-1-lg, dilution 1:100) diluted in blocking solution for 8-10 hours at 4 °C, washed three times with PBS and then incubated in the second antibody (Goat Anti-Rabbit IgG H&L, FITC, ZSGB-BIO, ZF-0311, dilution 1:100; Goat Anti-Mouse IgG H&L, Alexa Fluor® 594,ZSGB-BIO, ZF-0513, dilution 1:100) in blocking solution for 1 h. The cells were then washed three times with PBS. DAPI (Beyotime, C1005) was added for DNA staining 5min, the cells were then washed three times with PBS. Images were taken with a Zeiss LSM Confocal Microscope (Carl Zeiss, Jena, Germany).

**REFERENCES**

(1) Dai, Z.; Yu, M.; Yi, X.; Wu, Z.; Tian, F.; Miao, Y.; Song, W.; He, S.; Ahmad, E.; Guo, S.; et al. Chain-Length- and Saturation-Tuned Mechanics of Fluid Nanovesicles Direct Tumor Delivery. *ACS nano* **2019**, *13*, 7676-7689.

(2) Fu, Y.; Mackowiak, B.; Feng, D.; Lu, H.; Guan, Y.; Lehner, T.; Pan, H.; Wang, X. W.; He, Y.; Gao, B. MicroRNA-223 attenuates hepatocarcinogenesis by blocking hypoxia-driven angiogenesis and immunosuppression. *Gut* **2023**, 72, 1942-1958.


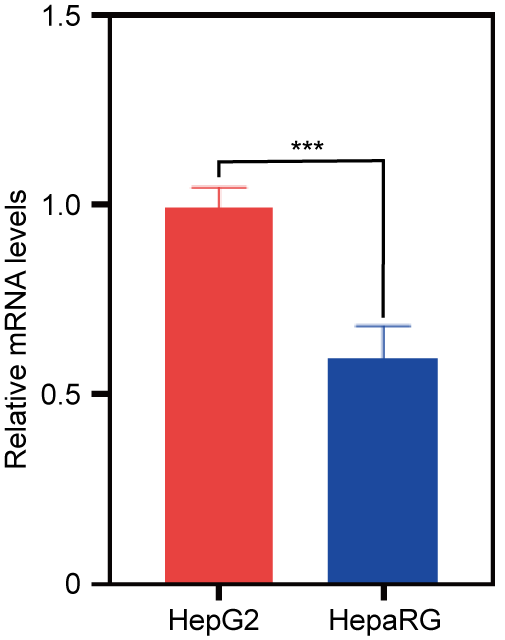
**Supplementary Figures and Tables**

**Figure S1.** mRNA expression of ASGP-receptor in HepG2 and HepaRG cells (n=3). Statistical significances were calculated via the Student’s t test (****p* < 0.001).

**
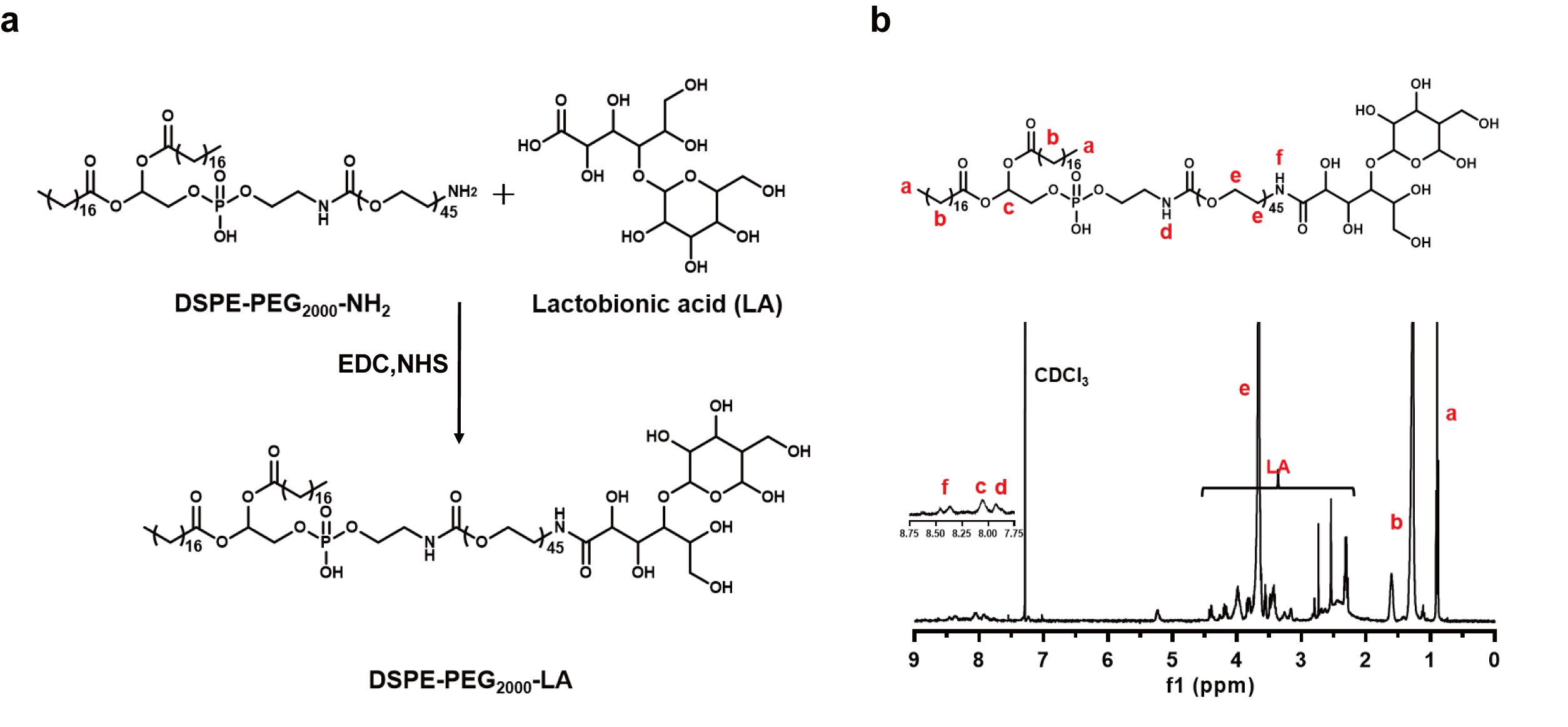
**

**Figure S2.** Synthesis of DSPE-PEG-LA and 1HNMR spectra of DSPE-PEG-LA. (a) Synthesis of DSPE-PEG-LA. (b) 1HNMR spectra of DSPE-PEG-NH2 and LA. The characteristic peaks LA was shown at 2.0-4.5 ppm, and that of DSPE-PEG2000-NH2 was shown at 7.75-8.75 ppm. The characteristic peaks of DSPE-PEG2000-NH2 and LA were present in DSPE-PEG-LA conjugates.


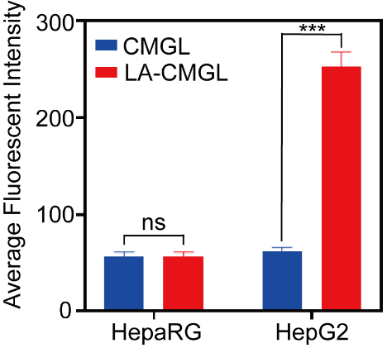
**Figure S3.** Mean fluorescence intensity of LA-CMGL in HepG2 and HepaRG detected by FCM. HepaRG cell was used as a negative control (n = 3). Data are mean ± SD. Statistical significances were calculated via the Student’s t test (*** *p* < 0.001).


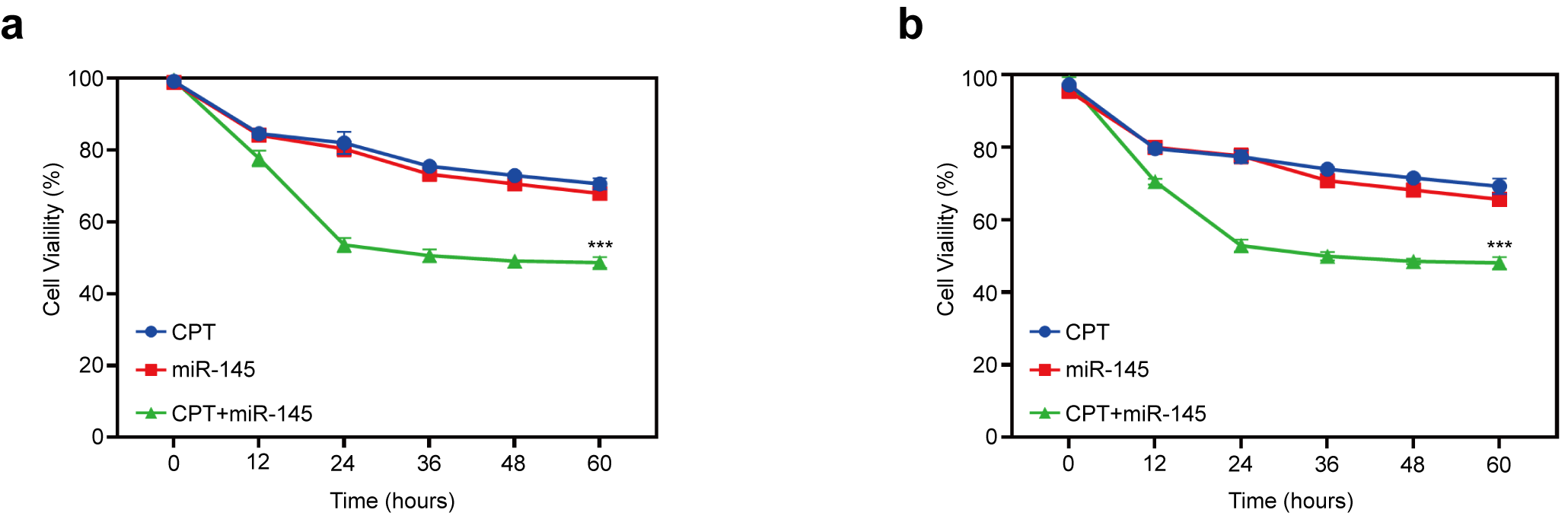
**Figure S4.** In vitro antitumor effect in Huh7 and Hep3B. Cell viability of Huh7 (a) and Hep3B (b) cells incubated with CPT, miR-145, CPT + miR-145 from 12 to 60 h (n = 3). Statistical significance was calculated via the one-way ANOVA with Tukey’s post hoc test (****p* < 0.001).


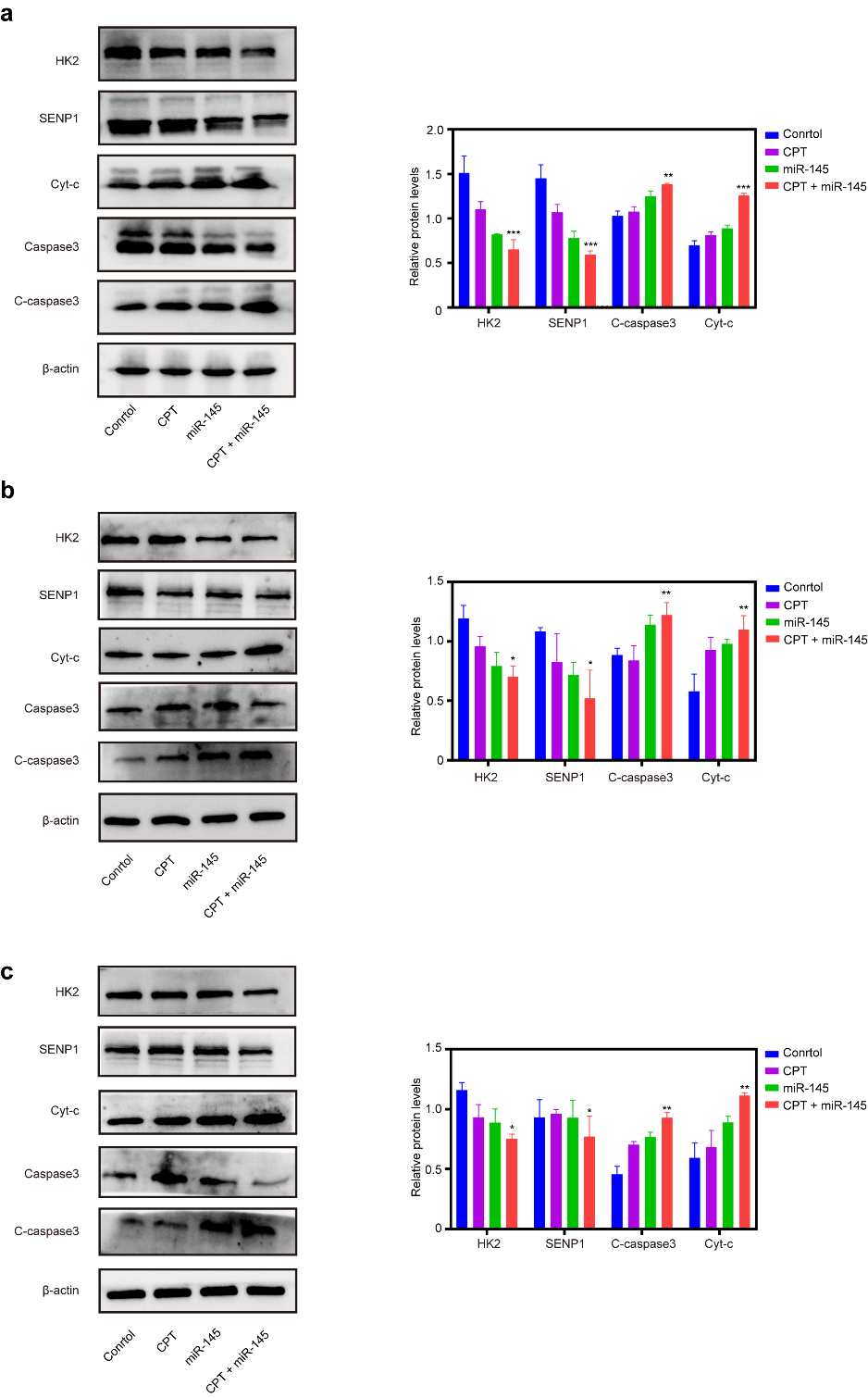
**Figure S5.** Synergetic antitumor mechanism of CPT and miR-145. Protein expression and quantification of HK2, SENP1, Cyt-c, C-Caspase3 and Caspase3 after miR-145 transfection in HepG2 (a), Huh7 (b) and Hep3B (c) cells. Data represent mean ± SD (n = 3). Statistical significance was calculated via the Student’s t-test (****p* < 0.001, ***p* < 0.01).


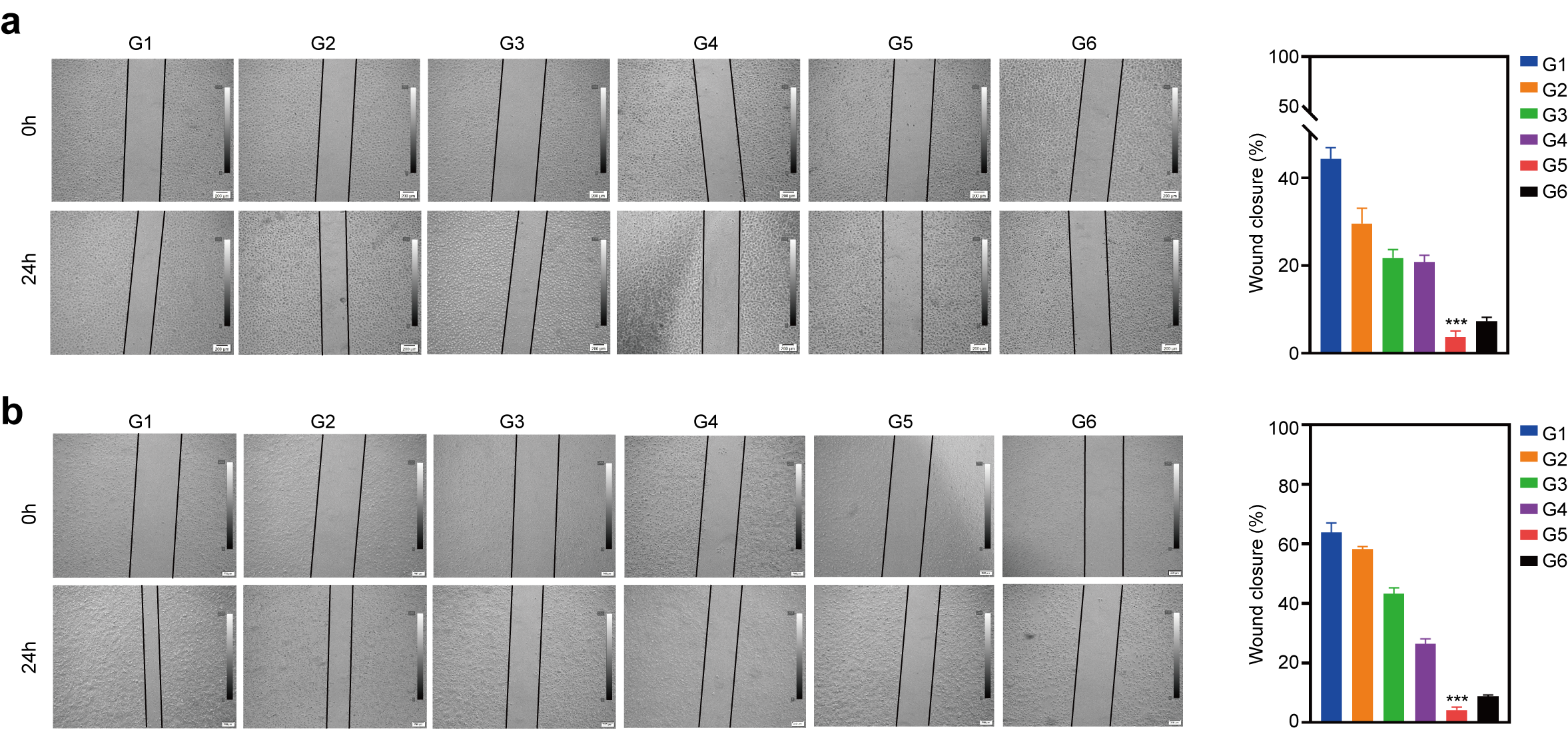
**Figure S6.** *In vitro* wound scratch assay of the Huh7 (a) and Hep3B (b) cells (n = 3). Statistical significance was calculated via the Student’s t-test (****p* < 0.001).


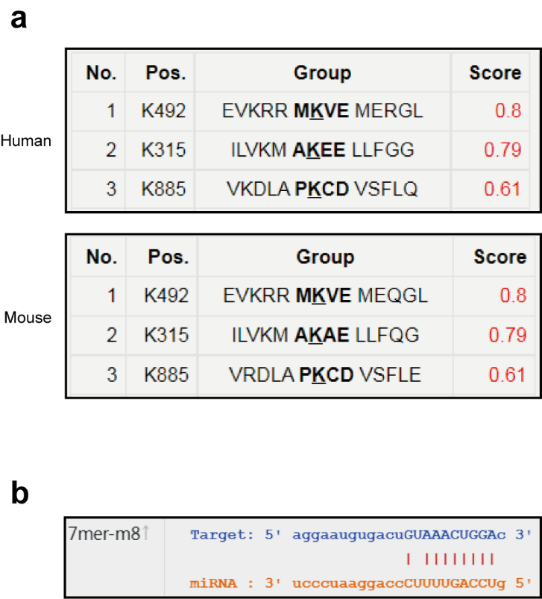
**Figure S7.** Potential sumo-ization sites of HK2 in different species.


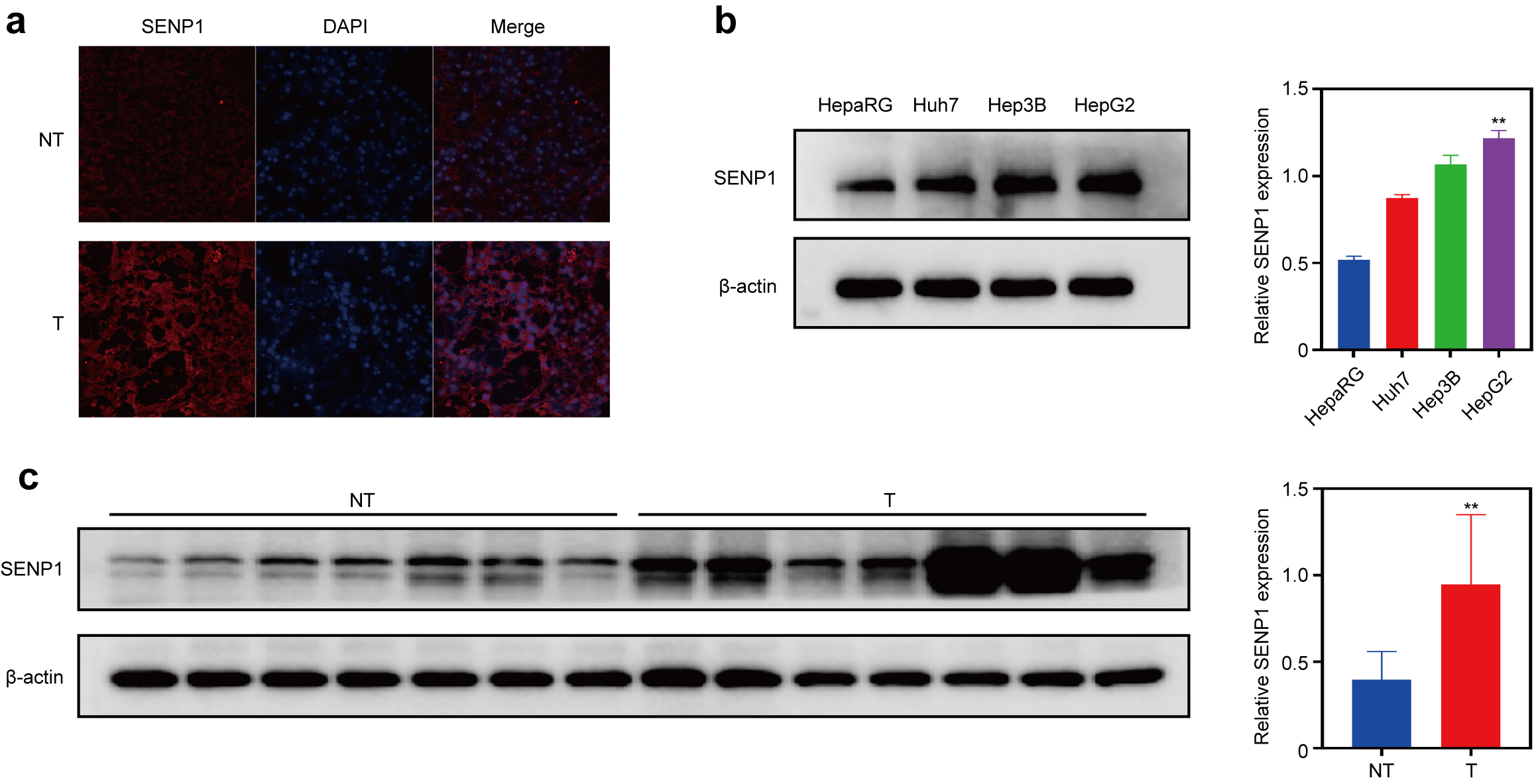
**Figure S8**. The expression of SENP1 in hepatocellular carcinoma. Immunofluorescence double staining of SENP1 (red) and DAPI (blue) in frozen sections of clinical HCC samples. (b) SENP1 protein expression in liver and hepatoma cell lines. (c) Expression of SENP1 protein in T (tumor) group and NT (Non-tumor) group corresponding to (a). Statistical significance was calculated via the Student’s t-test (***p* < 0.01).


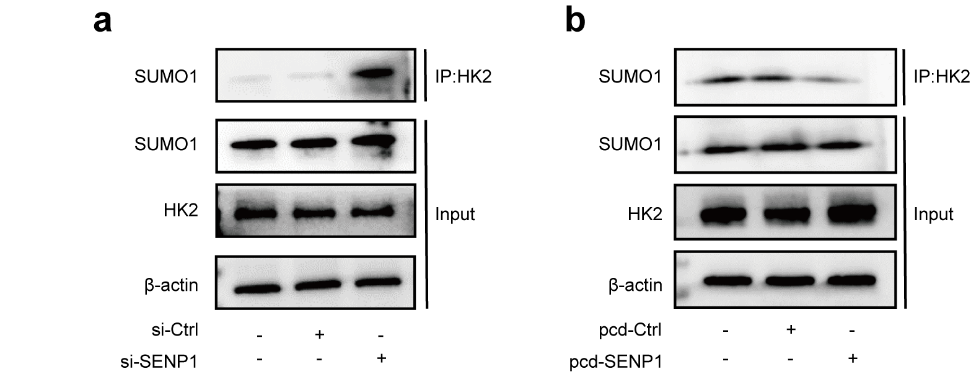

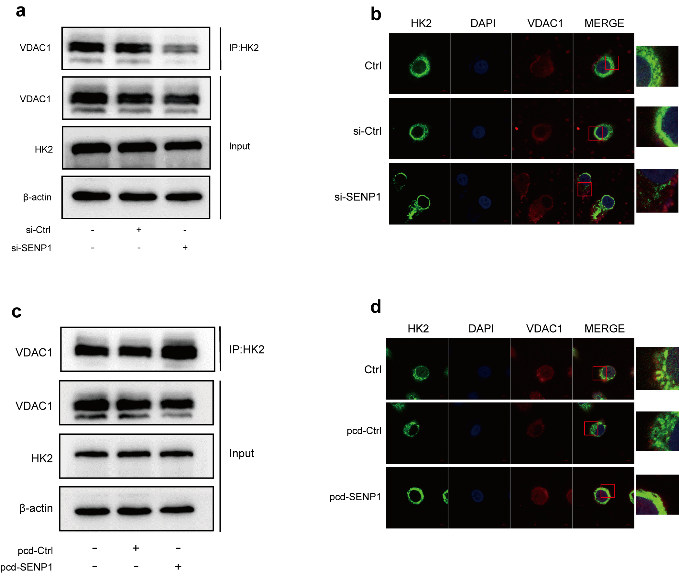
**Figure S9.** Co-IP of HK2 with SUMO1. (a) siSENP1 increased HK2 binding to SUMO1 after siSENP1 transfection. (b) pcdSENP1 decreased HK2 binding to SUMO1.

**Figure S10.** Co-localization of HK2 and VDAC1. (a) COIP of HK2 with VDAC1 after siSENP1 transfection. (b) Immunofluorescence staining of HK2 (green) and VDAC1 (red) co-localization after HepG2 transfection with siSENP1. (c) COIP of HK2 with VDAC1 after pcdSENP1 transfection. (d) Immunofluorescence staining of HK2 (green) and VDAC1 (red) co-localization after HepG2 transfection with pcdSENP1.


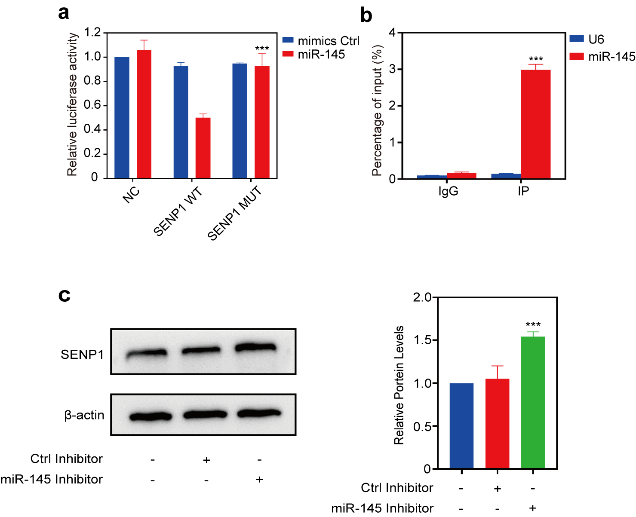

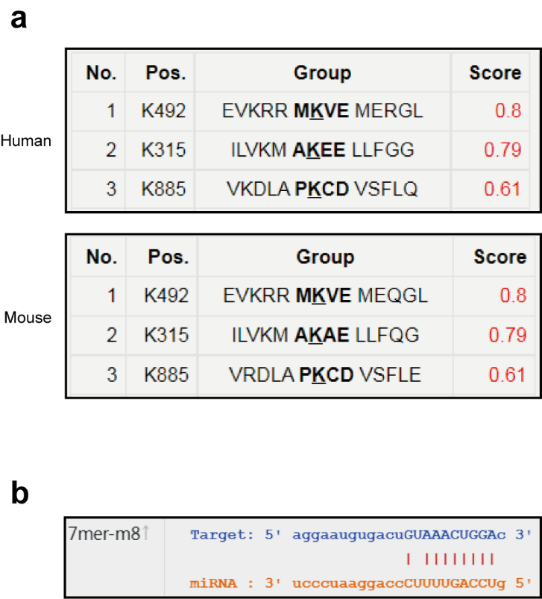
**Figure S11.** Bioinformatics approach to predict SENP1 as a target gene of miR-145.

**Figure S12.** miR-145 targets SENP1. (a) Dual luciferase report shows SENP1 is a target of miR-145. (b) RIP experiment to identify the correlation between SENP1 and miR-145. (c) miR-145 inhibitor promoted the expression of SENP1. Statistical significance was calculated via the Student’s t-test (****p* < 0.001).


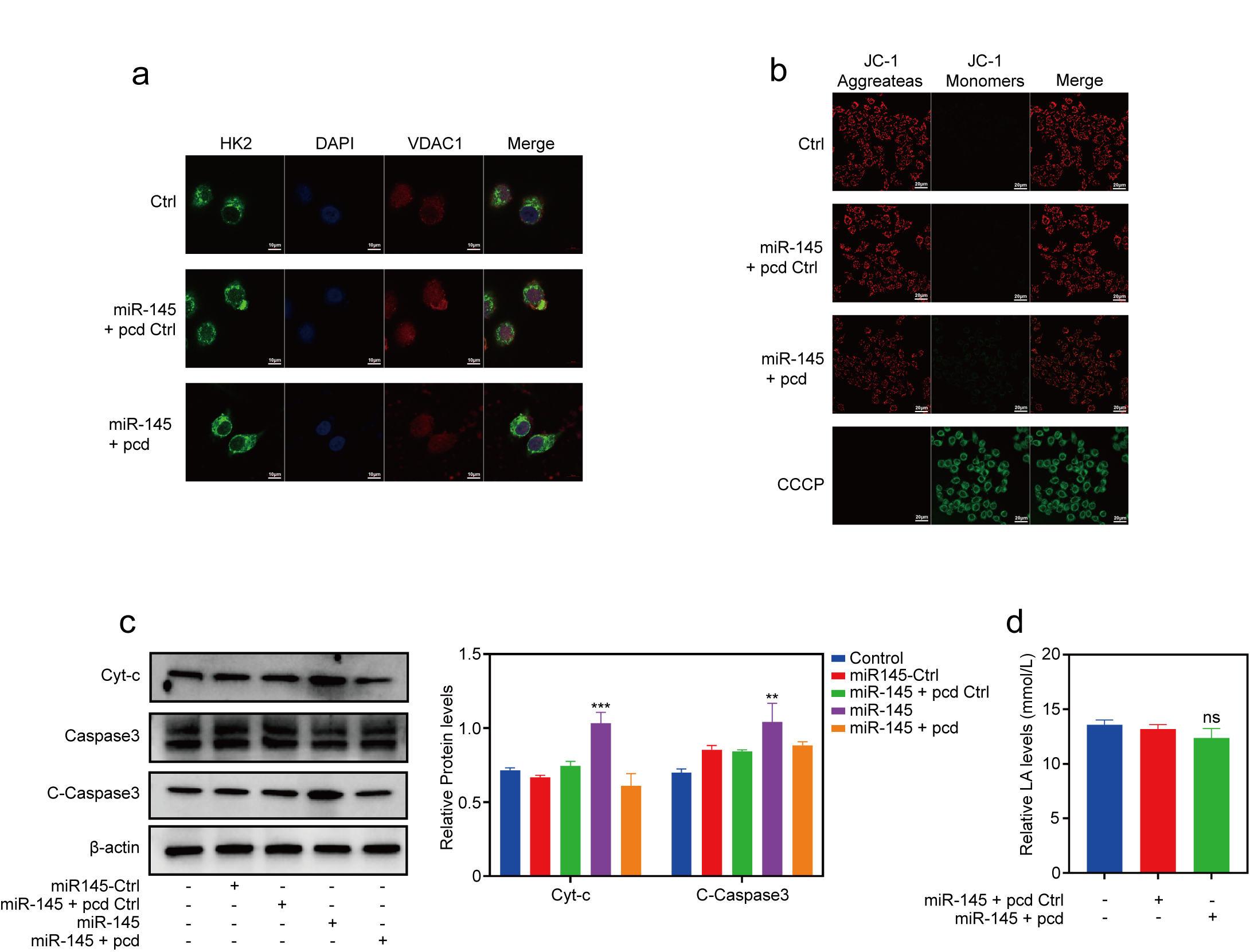
**Figure S13.** miR-145 regulates SENP1-mediated SUMOylation of HK2 in HepG2 cells. (a) Immunofluorescence staining of HK2 (green) and VDAC1 (red) co-localization after co-transfection. (b) Detection of mitochondrial membrane potential after co-transfection. (c) Protein expression of Cyt-c, C-Caspase3 and Caspase3 after co-transfection. (d) Lactate content of medium after co-transfection. Statistical significance was calculated via the Student’s t-test (^ns^ *p* > 0.05, ****p* < 0.001, ***p* < 0.01).


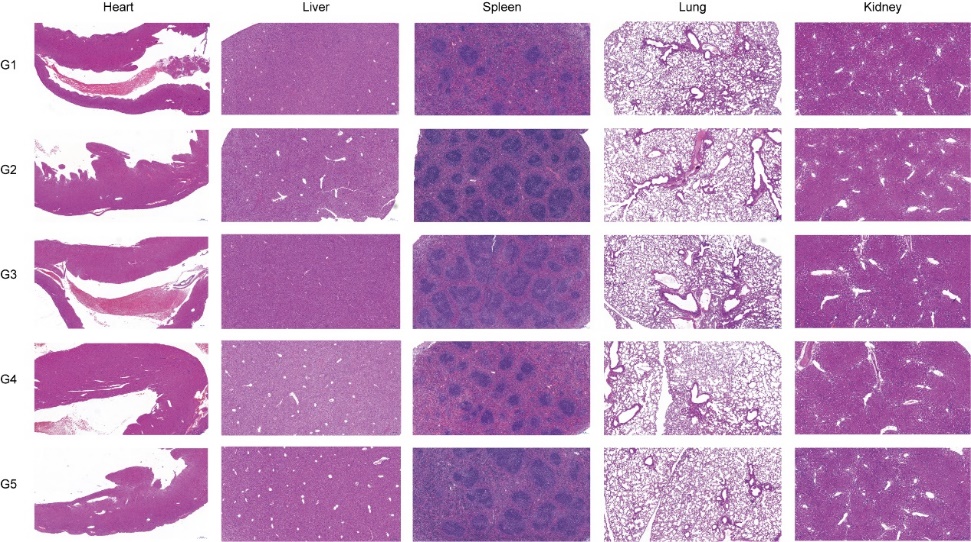


**Figure S14.** H&E stainiing sections of main organs (heart, liver, spleen, lung, and kidney) showed almost no obvious pathological abnormity of different treatment groups compared with PBS group (scale bar = 200 μm).


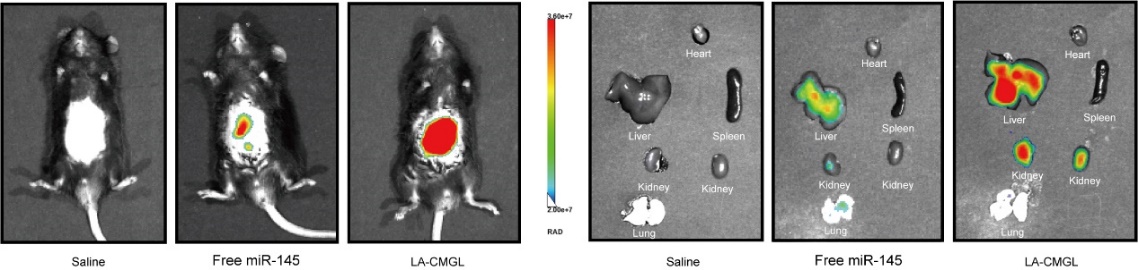


**Figure S15.** *In vivo* and *ex vivo* fluorescence imaging of major organs from normal mice after intravenous injection of saline, free miR-145 and LA-CMGL. LA-CMGL exhibited significantly higher fluorescence than free miR-145 at 6 h.

**Table S1.** Characteristics of Different Lipid Nanoparticles Formulations (mean ± SD, n=3).


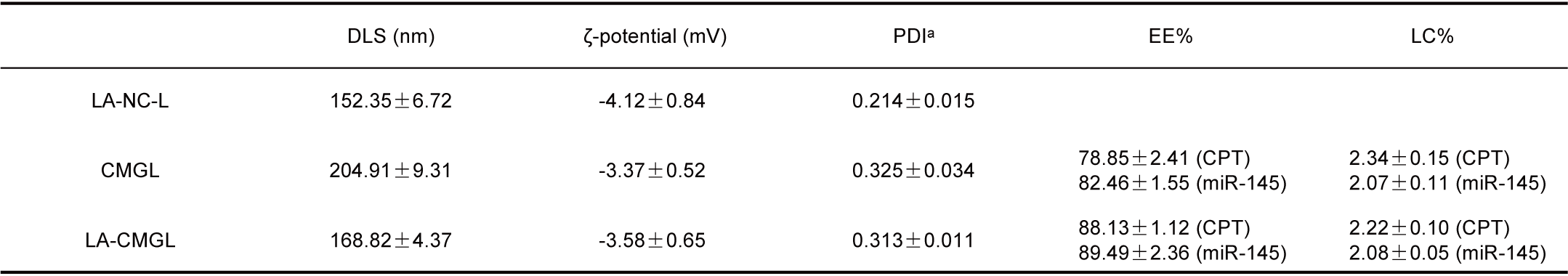


**Table S2.** RT-PCR Primer Sequences.


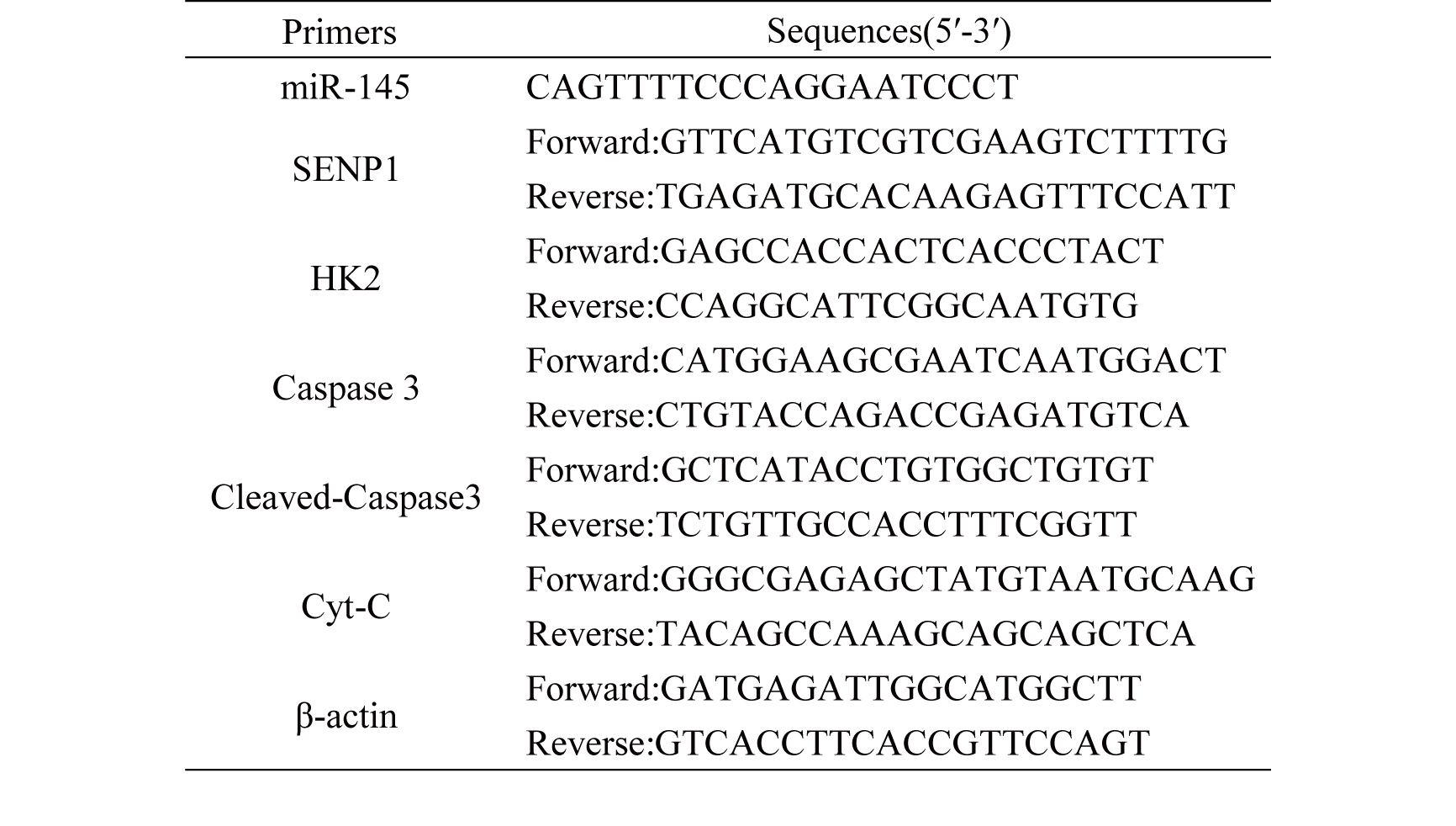

Supplement: Supplementary file 1 — Supplementary Material 1 [file 13046_2024_3167_MOESM1_ESM.doc]
